# Phosphoproteomic of the acetylcholine pathway enables discovery of the PKC-β-PIX-Rac1-PAK cascade as a stimulatory signal for aversive learning

**DOI:** 10.1038/s41380-022-01643-2

**Published:** 2022-06-03

**Authors:** Yukie Yamahashi, You-Hsin Lin, Akihiro Mouri, Sho Iwanaga, Kazuhiro Kawashima, Yuya Tokumoto, Yo Watanabe, Md. Omar Faruk, Xinjian Zhang, Daisuke Tsuboi, Takashi Nakano, Naoaki Saito, Taku Nagai, Kiyofumi Yamada, Kozo Kaibuchi

**Affiliations:** 1 Division of Cell Biology, International Center for Brain Science, Fujita Health University, 1-98 Dengakugakubo, Kusukake-cho, Toyoake, Aichi 470-1192 Japan; 2 Department of Cell Pharmacology, Nagoya University Graduate School of Medicine, 65 Tsurumai, Showa, Nagoya, 466-8550 Japan; 3 Department of Regulatory Science for Evaluation and Development of Pharmaceuticals and Devices, Fujita Health University, Graduate School of Health Sciences, 1-98 Dengakugakubo, Kutsukake-cho, Toyoake, Aichi 470-1192 Japan; 4 Division of Behavioral Neuropharmacology, International Center for Brain Science, Fujita Health University, 1-98 Dengakugakubo, Kutsukake-cho, Toyoake, Aichi 470-1192 Japan; 5 Department of Computational Biology, School of Medicine, Fujita Health University, 1-98 Dengakugakubo, Kutsukake-cho, Toyoake, Aichi 470-1192 Japan; 6 Laboratory of Molecular Pharmacology, Division of Signal Functions, Biosignal Research Center, Kobe University, 1-1 Rokkodai-cho, Nada, Kobe, Hyogo 657-8501 Japan; 7 Department of Neuropsychopharmacology and Hospital Pharmacy, Nagoya University Graduate School of Medicine, 65 Tsurumai, Showa, Nagoya, 466-8560 Japan

**Keywords:** Neuroscience, Molecular biology, Biochemistry

## Abstract

Acetylcholine is a neuromodulator critical for learning and memory. The cholinesterase inhibitor donepezil increases brain acetylcholine levels and improves Alzheimer’s disease (AD)-associated learning disabilities. Acetylcholine activates striatal/nucleus accumbens dopamine receptor D2-expressing medium spiny neurons (D2R-MSNs), which regulate aversive learning through muscarinic receptor M1 (M1R). However, how acetylcholine stimulates learning beyond M1Rs remains unresolved. Here, we found that acetylcholine stimulated protein kinase C (PKC) in mouse striatal/nucleus accumbens. Our original kinase-oriented phosphoproteomic analysis revealed 116 PKC substrate candidates, including Rac1 activator β-PIX. Acetylcholine induced β-PIX phosphorylation and activation, thereby stimulating Rac1 effector p21-activated kinase (PAK). Aversive stimulus activated the M1R-PKC-PAK pathway in mouse D2R-MSNs. D2R-MSN-specific expression of PAK mutants by the Cre-Flex system regulated dendritic spine structural plasticity and aversive learning. Donepezil induced PAK activation in both accumbal D2R-MSNs and in the CA1 region of the hippocampus and enhanced D2R-MSN-mediated aversive learning. These findings demonstrate that acetylcholine stimulates M1R-PKC-β-PIX-Rac1-PAK signaling in D2R-MSNs for aversive learning and imply the cascade’s therapeutic potential for AD as aversive learning is used to preliminarily screen AD drugs.

## Introduction

Approximately 45 years have passed since the correlation between decreased acetylcholine (ACh) levels in the brains of Alzheimer’s disease (AD) patients and learning disabilities identified the importance of the neuromodulator ACh in learning and memory [1, 2]. Indeed, ACh recovery in the brains of AD patients by the central cholinesterase inhibitor donepezil improves memory loss and learning disabilities [3, 4]. The role of ACh in learning has been examined extensively in the striatum/nucleus accumbens (NAc) [5–9], the brain region that is pivotal for emotional learning [10] and shows the highest ACh level [11]. The involvement of ACh in aversive learning has been demonstrated based on evidences that aversive stimuli, such as an electric foot shock, increase striatal/accumbal ACh levels [6, 12]. Furthermore, ACh has been shown to stimulate aversive learning in pharmacological studies [3, 13, 14]. However, the intracellular ACh signaling that regulates learning remains unresolved, which has limited the development of therapeutic strategies for CNS disorders with learning deficits, including AD [15].

In the striatum/NAc, ~95% of neurons are medium spiny neurons (MSNs), and 1–2% are cholinergic interneurons, the main source of local ACh [11]. MSNs are subdivided into dopamine receptor D1-expressing MSNs (D1R-MSNs) and dopamine receptor D2-expressing MSNs (D2R-MSNs) [16], which are involved in reward and aversive learning. The expression of reward and aversive learning depends on the balance between D1R-MSN activity and D2R-MSN activity, which are regulated by various neuromodulators [10, 17]. ACh is known to preferentially enhance D2R-MSN excitability through Gq-coupled receptor muscarinic receptor M1 (M1R), the only muscarinic receptor expressed on these MSNs [18, 19]. Regardless of M1R expression, ACh has an inhibitory effect on D1R-MSNs because of the expression of the Gi-coupled receptor M4R [19], which has a higher ACh affinity than M1R [20].

M1R primarily uses phospholipase C and protein kinase C (PKC) as downstream effectors for its signal transduction [21, 22]. To date, ACh intracellular signaling beyond M1R in the brain, including accumbal D2R-MSNs that enhance aversive learning, remains unelucidated due to the lack of methods for exploring downstream phosphorylation events. We have previously developed a novel phosphoproteomic method called kinase-oriented substrate screening (KIOSS), which uses affinity beads coated with phospho-Ser/Thr binding protein 14-3-3 to enrich phosphorylated proteins [23, 24], and identified more than 100 substrate candidates of PKA and MAPK downstream of dopamine-D1R [25]. We also found that dopamine-D1R signaling stimulated reward-related behavior and learning though the phosphorylation of Rasgrp2 by PKA and Npas4 by MAPK in D1R-MSNs [25, 26].

In this study, we employed D2R-MSN-mediated aversive learning as a model system to examine intracellular ACh signaling, which regulates learning ability. To explore downstream signaling of ACh, we used the KIOSS approach and identified 116 PKC substrate candidates. They include the Rho family GTPase Rac activator β-PIX, which forms a β-PIX/Git1/Rac1/p21-activated kinase (PAK) signaling complex that activates PAK and concomitantly regulates spine morphogenesis [27, 28], a critical process for learning. We found that ACh stimulated PAK activation through M1R and PKC ex vivo. Donepezil and aversive stimulus activated the M1R-PKC-PAK pathway in D2R-MSNs in vivo. PAK regulated dendritic spine structural plasticity and D2R-MSN-mediated aversive learning. Donepezil enhanced aversive learning through PAK in D2R-MSNs.

## Materials and methods

### Animals

C57BL/6 (RRID: IMSR_JAX:000664) mice and Drd1a-tdTomato [B6. Cg-Tg(Drd1a-tdTomato)6Calak/J, RRID: IMSR_JAX:016204] mice on the C57BL/6 background were purchased from Japan SLC (Shizuoka, Japan) and the Jackson Laboratory (Bar Harbor, ME, USA), respectively. Drd2-mVenus transgenic mice [C57BL/6J-Tg (Drd2-YFP) 364-5Koba/KobaRbrc, RRID: IMSR_RBRC02332] were generated as previously described [25]. Drd1a-Cre mice on a C57BL/6 background [B6. FVB (Cg)-Tg (Drd1a-cre) EY262Gsat/Mmucd, RRID: MMRRC_030989-UCD] and Adora2a-Cre mice on a C57BL/6 background [B6. FVB (Cg)-Tg (Adora2a-cre) KG139Gsat/Mmucd, RRID: MMRRC_036158-UCD] was provided by the Mutant Mouse Research and Resource Center (MMRRC) at UC Davis (Davis, CA, USA).

Heterozygous Drd1a-Cre and Adora2a-Cre male mice were obtained by crossing homozygous Drd1a-Cre and Adora2a-Cre male mice with C57BL/6 female mice. Drd1-tdTomato/Drd2-mVenus double transgenic mice were generated by crossing heterozygous Drd1a-tdTomato mice with homozygous Drd2-mVenus mice. Double transgenic mice were identified by PCR with genomic DNA prepared from tail clips. Genotyping primers were:

### Drd1-tdTomato

forward: 5′-CTTCTGAGGCGGAAAGAACC-3′

reverse: 5′-CGGCAAACGGACAGAAGCATT-3′

### Drd2-mVenus

forward: 5′-CAGTATCTATTATTTCTTTTAGAACG-3′

reverse: 5′-GCAGATTAACTTCAGGGTCAGC-3′

Mice were housed under a standard 12-h light/dark cycle (light phase 9:00–21:00) at a constant temperature of 23 ± 1 °C with free access to food and water throughout the experiments. The male mice used in this study were 7–12 weeks old and had a body weight of 22–29 g. All animal experiments were approved and performed in accordance with the guidelines for the care and use of laboratory animals established by the Animal Experiments Committee of Nagoya University Graduate School of Medicine (reference number: M210568-002) and Fujita Health University (reference number: AP20037). All experiments were conducted in compliance with the ARRIVE guidelines.

### Cell lines

293AAV (RRID: CVCL_KA64) (Cell BioLabs, San Diego, CA, USA) cell lines were cultured in Dulbecco’s modified Eagle’s medium (SIGMA-Aldrich, St. Louis, MO, USA) containing 10% fetal bovine serum (SAFC Biosciences, Lenexa, KS, USA). Cells were grown in a humidified atmosphere of 5% CO_2_ at 37 °C. The cell lines used in this study are not listed as a commonly misidentified cell line by ICLAC. Cells were not authenticated during this study and were passaged a maximum of ten times. Cells were tested negative for mycoplasma contamination by PCR method.

### Antibodies

The following antibodies were used for immunoblotting: rabbit polyclonal anti-phospho PAK1 (S144) (RRID:AB_2299279, #2606S, 1:1000), rabbit polyclonal anti-phospho NMDAR1 (S890) (RRID:AB_2294781, #3381S, 1:1000), rabbit polyclonal anti-PAK1 (RRID:AB_330222, #2602S, 1:1000), rabbit monoclonal anti-phospho cofilin (S3) (RRID: AB_2080597, #3313S, 1:1000) (Cell Signaling Technology, Inc, Danvers, MA, USA); mouse monoclonal anti-cofilin (RRID:AB_11043339, #66057-1-Ig, 1:1000) (Proteintech, Rosemont, IL, USA); mouse monoclonal anti-LIMK1 (RRID:AB_ 2723320, #MA5-25569, 1:1000) (Thermo Fisher Scientific, Waltham, MA, USA); rabbit polyclonal anti-phospho LIMK1 (T508) (RRID:AB_10621739, #SAB4300103, 1:1000) (Sigma-Aldrich); mouse monoclonal anti-NMDAR1 (RRID:AB_10002447, #NB300-118, 1:2000) (Novus Biologicals, Littleton, CO, USA); mouse monoclonal anti-GST (RRID: AB_2883970, #017-21854, 1:1000), mouse monoclonal anti-β-Actin (RRID: AB_2858279, #010-27841, 1:2000) (Fujifilm Wako, Osaka, Japan); mouse monoclonal anti-BetaPIX (RRID:AB_399166, # 611648, 1:1000) (BD Biosciences, San Jose, CA, USA); rabbit polyclonal anti-phospho BetaPIX (T76), (1:500) (kindly provided by NS, Kobe University, Kobe, Japan); goat anti-rabbit IgG Alexa Fluor 680 (RRID:AB_2535758, #A-21109, 1:20,000) (Thermo Fisher Scientific); and goat anti-mouse IgG DyLight conjugate 800 (RRID:AB_10693543, #5257P, 1:20,000) (Cell Signaling Technology).

The following antibodies were used for immunohistochemistry: rabbit polyclonal anti-phospho PAK1 (S144) antibody (RRID: AB_2533801, #44-940G, 1:300) (Thermo Fisher Scientific); rabbit polyclonal anti-phospho BetaPIX (T76), (1:300) (kindly provided by NS, Kobe University, Kobe, Japan); rat monoclonal anti-GFP antibody (RRID:AB_10013361, #04404-84, 1:500) (Nacalai Tesque, Kyoto, Japan); mouse monoclonal NeuN antibody (RRID: AB_2298772, #MAB377, 1:1000) (Millipore, Burlington, MA, USA) CF405S-conjugated donkey anti-rabbit IgG secondary antibody (RRID: AB_2860031, #20420, 1:500) (Biotium, Hayward, CA, USA); Alexa Fluor 488-conjugated donkey anti-rat IgG (RRID: AB_2535794, #A-21208, 1:500), Alexa Fluor 488-conjugated donkey anti-rabbit IgG (RRID: AB_2535792, #A-21206, 1:500), and Alexa Fluor 555-conjugated donkey anti-mouse IgG (RRID: AB_2536180, # A-31570, 1:500) secondary antibodies (Thermo Fisher Scientific).

### Drugs

The following drugs were used in the present study: the M1R inhibitor VU0255035 and the PKC activator PEP-005 (Abcam, Cambridge, UK); the PKC inhibitor GF109203X (Tocris Bioscience, Bristol, UK); the PKC inhibitor NPC-15437 (Signal Chem Pharmaceuticals, Richmond, BC, Canada); the PAK inhibitor FRAX486 (AdooQ BioScience, Irvine, CA, USA); carbachol and donepezil hydrochloride (Sigma-Aldrich, St. Louis, MO, USA); and the M1R activator VU0364572 TFA salt (Glixx lab, Hopkinton, MA, USA).

For ex vivo experiments, carbachol and VU0364572 were dissolved in Milli-Q grade water. VU0255035, PEP-005, GF109203X, and FRAX486 were dissolved in DMSO (Fujifilm Wako). Striatal slices were pretreated with 10 μM GF109203X for 90 min. For in vivo experiments, VU0255035 was dissolved in a 5% (v/v) solution of lactic acid in saline, and the pH was adjusted to 6.5–7.0 with a dilute NaOH solution [29]. FRAX486 was dissolved in a 20% (wt/vol) solution of hydroxypropyl-β-cyclodextrin (Sigma-Aldrich) in saline [30]. Donepezil hydrochloride, NPC-15437 [31], and VU0364572 [32] were dissolved in saline.

Donepezil hydrochloride (0.4 mg/kg), FRAX 486 (20 mg/kg or 40 mg/kg), and NPC-15437 (1 mg/kg) were administered via subcutaneous injection at the neck scruff. VU0364572 (25 mg/kg) and VU0255035 (20 mg/kg or 40 mg/kg) were administered via intraperitoneal injection. The compounds were administered at the following times before passive avoidance training: donepezil 30 min; FRAX 486 6 h; VU0255035 25 min, NPC-15437 30 min.

### Plasmid constructs

To generate an AAV vector that bicistronically expresses the EGFP protein and the target protein under the control of the CAMKII promoter in a Cre-dependent manner, we constructed a pAAV-CAMKII-Flex-EGFP-P2A-MCS-WPRE plasmid. Specifically, the EGFP cDNA sequence, followed by a self-cleaving 2A peptide sequence and a multiple cloning site (MCS), was cloned into the MCS of the pAAV-CAMKII-Flex-MCS-WPRE plasmid in an inverted manner. Dominant negative (DN) Rac, DN PAK, and constitutively active (CA) PAK cDNAs were subcloned into the pAAV-CAMKII-Flex-EGFP-P2A-MCS-WPRE plasmid. The cDNA of DN Rac was obtained as previously described [33]. The cDNAs encoding DN PAK and CA PAK were obtained by PCR amplification of the autoinhibitory domain of mouse PAK1 (83 aa-149 aa) [34] using primers (forward: 5′-CGGGATCCATGCATACAATTCATGTTGGTTTTG-3′; reverse: 5′-CGGAATTCTTATGACTTATCTGTAAAACTCATG-3′) and the catalytic domain of mouse PAK1 (233 aa–544 aa) [35] using primers (forward: 5′-CGGGATCCATGGCTTTGACCCGGAACACGGAAAAACAG; reverse: 5′-CCGCTCGAGTCAGTGATTGTTCTTGGTTGCCTCTTTTG-3′) from mouse brain cDNAs.

### Preparation and incubation of striatal slices

Striatal slices were prepared as described previously [36] with some modifications. Male C57BL/6 mice were decapitated, and the brains were rapidly removed. Coronal brain slices (350 μm) were prepared using a VT1200S vibratome (Leica Microsystems, Wetzlar, Germany) in ice-cold, oxygenated Krebs-HCO_3_− buffer (124 mM NaCl, 4 mM KCl, 26 mM NaHCO_3_, 1.5 mM CaCl_2_, 1.25 mM KH_2_PO_4_, 1.5 mM MgSO_4_, and 10 mM D-glucose, pH 7.4). After the striatum/NAc were dissected from the brain slices, striatal/accumbal slices were incubated at 30 °C in Krebs-HCO_3_− buffer containing 10 μg/ml adenosine deaminase (Roche, Basal, Switzerland) for 30 min under constant oxygenation with 95% O_2_/5% CO_2_. The buffer was then replaced with fresh Krebs-HCO_3_− buffer and preincubated for 30 min. Striatal/accumbal slices were pretreated with the indicated inhibitors and were then stimulated with the indicated activators. After stimulation, the slices were snap frozen in liquid nitrogen and stored at −80 °C until assayed. The slices were lysed in lysis buffer [1% SDS, 1 mM EDTA, 1 mM DTT, 1% glycerol, 50 mM Na_2_HPO_3_, phosphatase inhibitor calyculin A (50 nM) (Fujifilm Wako), protease inhibitor cocktail (Roche), pH 7.0] and sonicated for 20 s. Lysates were immediately heated at 70 °C for 10 min and briefly centrifuged at 15,000 rpm for 1 min to remove debris. The protein concentration of lysates was determined by BCA assays (Fujifilm Wako).

### In vivo sample preparation

In vivo sample preparation for immunoblot analysis was carried out as previously described [37]. After drug administration or AAV injection, mice were decapitated. Mouse heads were immediately immersed in liquid nitrogen for 4 s, and the whole brains were rapidly removed. The brains were coronally sectioned (~ 2 mm) using a mouse brain slicer matrix (#MBS-S1C, Brain Science Idea Co., Ltd., Osaka, Japan) chilled on ice. The NAc was collected using a 2 mm diameter biopsy punch (Kai corporation, Tokyo, Japan) and dorsal region of hippocampi were collected using a 1.5 mm diameter biopsy punch (Kai corporation) on an ice-cold plate. The tissue sample was snap frozen in liquid nitrogen and stored at −80 °C until immunoblotting.

### Mass spectrometry

Sample preparation for mass spectrometry was carried out as previously described [25]. Briefly, brain slices were lysed in RIPA buffer [50 mM Tris/HCl, 1 mM EDTA, 150 mM NaCl, 1% NP-40, 0.5% sodium deoxycholate, 0.1% SDS, protease inhibitor cocktail (Roche), and PhosStop (Roche), pH 7.5] and sonicated for 30 s. After the lysates were adjusted to equal concentrations, the lysates were subjected to affinity chromatography using glutathione Sepharose 4B beads (GE Healthcare Bio-Sciences AB, Uppsala, Sweden) coated with GST-14-3-3 (500 pmol) for 60 min at 4 °C. The beads were washed with RIPA buffer three times and three more times with wash buffer (50 mM Tris-HCl, 1 mM EDTA, and 150 mM NaCl, pH 7.5). The bound proteins were extracted from the beads using guanidine solution (7 M guanidine and 50 mM Tris-HCl, pH 8.0), reduced via incubation in 5 mM dithiothreitol for 30 min, and alkylated using 10 mM iodoacetamide for 1 h in the dark. The proteins were digested with 0.5 μg of trypsin/Lys-C (Promega) in 50 mM NH_4_HCO_3_/1.2 M urea solution overnight at 37 °C. Phosphopeptide enrichment was carried out using a Titansphere Phos-TiO Kit (GL Sciences, Tokyo, Japan) according to the manufacturer’s instructions. Further demineralization was carried out using SPE c-tips (Nikkyo Technos, Tokyo, Japan) according to the manufacturer’s instructions. The peptides were analyzed by LC/MS/MS using an Orbitrap Fusion mass spectrometer (Thermo Fisher Scientific) as previously described [25].

A peak list was generated and calibrated using MaxQuant software (version 1.4.1.2) [38]. Database searches against the reference proteome of *Mus musculus* obtained from UniProtKB in March 2014 were performed using MaxQuant software. Fixed modification was set to cysteine carbamidomethylation, and the variable modifications were set to methionine oxidation, Ser/Thr/Tyr phosphorylation, and N-terminal acetylation. False discovery rates for the peptide, protein, and site levels were set to 0.01. Two missed cleavages by trypsin/Lys-C were allowed.

### Rac GEF affinity assay

GST-RacG15A protein purification and GST-RacG15A active GEF pull-down experiments were carried out as previously described [39, 40] with some modifications. Briefly, the expression of GST-RacG15A protein in *Escherichia coli* (BL21-pLys) was induced with 100 μM IPTG and induced for 16 h at 25 °C. GST-RacG15A was purified by the batch-binding method using MagneGST glutathione particles (Promega, Madison, WI, USA). GST-RacG15A-bound beads were washed with lysis buffer twice and wash buffer twice and were stored at −80 °C until use. Frozen mouse brain slices were lysed in lysis buffer [20 mM HEPES, pH 7.6, 1% Triton X-100, 150 mM NaCl, 5 mM MgCl_2_, 1 mM DTT, protease and phosphatase inhibitor cocktail (Roche)] and sonicated for 20 s. The lysates were centrifuged at 15,000 rpm for 1 min to remove debris. Lysates were adjusted to equal protein concentrations and incubated with 50 μg of GST-RacG15A immobilized on beads for 60 min at 4 °C. The beads were washed with lysis buffer three times and incubated in 30 μl of SDS sample buffer [41]. The eluted samples were boiled for 10 min at 70 °C and subjected to SDS-PAGE immunoblot analysis.

### SDS-PAGE immunoblot analysis

Laemmli SDS-PAGE was carried out using 10, 12, or 16% polyacrylamide gels. For all immunoblots, except pCofilin and Cofilin immunoblots, the proteins were transferred to 0.45 μm pore-size polyvinylidene difluoride membranes (Immobilon- FL, Merck). For pCofilin and Cofilin immunoblots, 0.22 μm pore-size nitrocellulose membranes (Bio-Rad, Hercules, CA) were used. The membranes were blocked for 1 h at RT with Blocking-One (Nacalai Tesque) or Blocking-One P (Nacalai Tesque) and were then incubated with primary antibodies overnight at 4 °C. The membranes were washed with 0.05% Tween/Tris-buffered saline (TBS) three times and incubated with goat anti-rabbit IgG Alexa Fluor 680 (RRID: AB_2535758, Thermo Fisher Scientific) and anti-mouse IgG DyLight 800 conjugate (RRID: AB_10693543, Cell Signaling Technology) secondary antibodies for 30 min at RT. Proteins were detected by using an infrared (LI-COR Biosciences, Lincoln, NE) imaging system. Band intensities were quantified using ImageStudio software (RRID: SCR_015795, LI-COR Biosciences).

### Immunohistochemical analysis

Mice were administered an anesthetic mixture of medetomidine (0.3 mg/kg, i.p.) (Nippon Zenyaku Kogyo, Koriyama, Fukushima, Japan), midazolam (4 mg/kg, i.p.) (Maruishi Pharmaceutical, Osaka, Japan), and butorphanol (5 mg/kg, i.p.) (Meiji Seika Pharma, Tokyo, Japan) and transcardially perfused with 4% paraformaldehyde (PFA) in phosphate-buffered saline (PBS). The whole brains were removed and immersed in 4% PFA in PBS overnight at 4 °C to postfix the brains. The brains were then cryoprotected in 30% sucrose by sequentially immersing them in 20% sucrose in PBS and 30% sucrose in PBS at 4 °C. The brains were frozen in optimal cutting temperature medium (Muto Pure Chemicals, Tokyo, Japan) and sectioned coronally by using a cryostat (Leica CM1850, Leica Microsystems, Wetzlar, Germany). For immunohistochemical staining of pPAK1 (S144), free floating immunohistochemistry was carried out (slice thickness: 25 μm). For immunohistochemical staining of pβ-PIX (T76), slide-mounted immunohistochemistry using adhesive glass slide (#MAS-01, Matsunami, Kishiwada, Japan) was carried out (slice thickness: 8 μm) to limit antibody solution.

Brain slices were immediately fixed in 4% PFA for 5 min at RT and incubated in 0.3% Triton X-100/TBS for 10 min to permeabilize the slices. After the slices were washed twice with TBS for 10 min, they were blocked with Blocking-One P for 8 h at 4 °C or 1 h at RT. The brain slices were stained with the indicated primary antibodies overnight at 4 °C [anti-pPAK1 (S144) antibody staining] or for 72 h at 4 °C [for anti-pβ-PIX (T76) antibody staining]. After the slices were washed with 0.1% Triton X-100/TBS for 10 min three times, they were stained with CF405S-conjugated donkey anti-rabbit IgG (Biotium) (RRID: AB_2860031) or Alexa Fluor 488-conjugated donkey anti-rat IgG (RRID: AB_2535794) (Thermo Fisher Scientific) secondary antibodies for 1 h at RT. Nuclei were visualized by staining with TO-PRO3 iodide (Thermo Fisher Scientific) or DAPI (Cell Signaling Technology). Confocal images were recorded with LSM 780 microscopes built around an Axio Observer Z1 with Plan-Apochromat 10 x (numerical aperture [NA] 0.45) or Plan-Apochromat 63 x (NA 1.40) lenses under the control of ZEN Digital Imaging for Light Microscopy (RRID: SCR_013672, Carl Zeiss, Oberkochen. Germany). The entire image of a coronal brain section was acquired by tile scanning. For quantification of the pPAK1 (S144)-positive cells and pβ-PIX (T76)-positive cells, images were acquired within a 300 μm × 300 μm region of interest in the NAc core. The number of target cells was counted by using ZEN 2012 (blue edition) software (RRID: SCR_013672, Carl Zeiss, Oberkochen. Germany).

### AAV preparation

AAV vectors were prepared and titered as described previously [25]. AAV plasmid, pHelper plasmid (Cell BioLabs), and pAAV-DJ plasmid (Cell BioLabs) were cotransfected into 293AAV cells. Seventy-two hours after transfection, cells were harvested. AAV vectors produced in 293AAV cells were purified by CsCl gradient ultracentrifugation using a SW41Ti rotor (Beckman-Coulter Life Science, Indianapolis, IN). The formed gradients were fractionated, and the AAV titer of each fraction was evaluated by qPCR (TOYOBO, Osaka, Japan). AAV-containing fractions were combined and subjected to a second round of CsCl gradient ultracentrifugation for further purification. After ultracentrifugation, the AAV titer of each fraction was evaluated by qPCR. AAV-rich fractions were combined and dialyzed using a dialysis cassette Slide-A-Lyzer (Thermo Fisher Scientific) to remove CsCl. The final AAV titer was determined by qPCR.

### AAV virus injection

AAV virus injection into the NAc was carried out as previously described [25] with some modifications. Mice were administered with an anesthetic mixture of medetomidine (0.3 mg/kg, i.p.) (Nippon Zenyaku Kogyo), midazolam (4 mg/kg, i.p.) (Maruishi Pharmaceutical), and butorphanol (5 mg/kg, i.p.) (Meiji Seika Pharma) and positioned in a stereotaxic frame (David Kopf, Tujunga, CA, USA) before performing surgery. AAV virus was injected into the NAc through a glass microinjection capillary tube at a 10° angle and at a rate of 0.1 μl/min.

For the step-through passive avoidance test, AAV virus (1.0 × 10^12^ genome copies/ml) was injected at four sites (0.5 μl/site) in the NAc. The anteroposterior, mediolateral, and dorsoventral coordinates relative to bregma were as follows: +1.6 mm, ±1.5 mm, and −4.4 mm; and +1.0 mm, ±1.6 mm, and −4.5 mm.

For dendritic spine analysis, AAV virus (3.0 × 10^11^ genome copies/ml) was injected at two sites (1.0 μl/site) in the NAc. The anteroposterior, mediolateral, and dorsoventral coordinates relative to bregma were as follows: +1.6 mm, ± 1.5 mm, and −4.4 mm. All experiments were performed 3 weeks after the injection.

### Step-through passive avoidance test

A step-through passive avoidance test was conducted as previously described [17]. The experimental apparatus consisted of two compartments (each 25 × 15 × 15 cm high), one illuminated and one dark, equipped with a grid floor. The two compartments were separated by a guillotine door. The entire test was composed of three sessions over three consecutive days (24 h apart). (1) In the habituation phase, the mouse was placed in the illuminated component. Fifteen seconds later, the door opened, and the mouse was given 2 min to enter the dark component. Once the mouse entered the dark component, the door closed, and the animal was removed. (2) In the training phase, the mouse was placed in the illuminated compartment with the door closed. Fifteen seconds later, the door opened, and the mouse was allowed to enter the dark component. Once the mouse entered the dark component with all four paws in, the door was closed, and an electric foot shock (0.4 mA, 2.0 s or 0.3 mA, 1.0 s) was delivered through the grid floor. The mouse was returned to the home cage 15 s later. (3) In the testing phase, the mouse was again placed in the illuminated compartment, and the door opened 15 s later. The latency to enter the dark component was recorded for up to 300 s. When the mice did not enter for at least 300 s, a score of 300 s was assigned.

### Novel-object recognition test

The novel-object recognition test was conducted as previously described [42]. The test procedure consisted of three sessions: habituation, training, and retention. Each mouse was individually habituated to a Plexiglas box (30 × 30 × 35 high cm) by being given 10 min exploration time in the box without any objects present for 3 days (habituation session). During the training session, two objects were placed in a back corner of the box. The objects were a golf ball, wooden cylinders, and square pyramids, which were different in shape and color but similar in size. A mouse was then placed midway toward the front of the box, and the total time it spent exploring the two objects was recorded for 10 min. A mouse was considered to be exploring the object when its head was facing the object or it was touching or sniffing the object. During the retention session, the mouse was placed back into the same box 24 h after the training session, but one of the familiar objects used during training was replaced with a novel object. The mouse was then allowed to explore freely for 10 min and the time spent exploring each object was recorded. Throughout the experiments, the objects were used in a counterbalanced manner in terms of their physical complexity and emotional neutrality. The preference index, a ratio of the amount of time spent exploring any one of the two objects (training session) or the novel object (retention session) over the total time spent exploring both objects, was used to measure cognitive function.

### Contextual fear conditioning test

The contextual fear conditioning tests were performed in accordance with the method outlined in a previous report [42]. For the conditioning phase, mice were placed in the conditioning cage (25 × 31 × 11 high cm). Then, 5 s of a foot shock of 0.6 mA was delivered from the metal rods of the grid floor through a shock generator (Brain Science idea Co. Ltd. Osaka, Japan). This shock was repeated four times at 15-s intervals. Contextual tests were carried out 1 day after fear conditioning. Mice were placed in the conditioning cage, and the freezing response was measured for 2 min in the absence of the conditioned stimulus.

### Y-maze test

The Y-maze test was conducted as previously described [42]. The maze was made of black painted wood; each arm was 40 cm long, 12 cm high, 3 cm wide at the bottom, and 10 cm wide at the top. The arms converged at an equilateral triangle that was 4 cm at its longest axis. Each mouse was placed at the center of the apparatus and allowed to move freely through the maze during an 8-min session. Arm entries were recorded by video camera. Alternation was defined as successive entry into the three different arms, with overlapping triplet sets counted. Alternation behavior (%) was calculated as the ratio of actual alternations to possible alternations (defined as the number of arm entries minus two), multiplied by 100.

### Dendritic spine imaging and analysis

Dendritic spine imaging was performed as previously described [25] with some modifications. Three weeks after mice were injected with AAV virus, mice were transcardially perfused with 4% PFA in PBS. The whole brains were removed and immersed in 4% PFA in PBS overnight at 4 °C to postfix the brains. The brains were sectioned coronally (100 μm) by using a VT1200S vibratome (Leica Microsystems, Wetzlar, Germany). The slices were then immunostained with GFP antibody by following the same procedures in the immunohistochemical analysis described above, with the exception that slices were washed with 0.01% Triton X-100/TBS after blocking, primary antibody staining, and secondary antibody staining. For spine morphology analysis, Z-stacks of secondary or tertiary dendrites (>20 μm long, >50 μm away from the soma) were captured with an LSM 780 confocal microscope using a ×63 objective (NA = 1.40, 5X zoom). The Z-dimensional increment was set to 0.3 μm, and the pinhole was set to 1 arbitrary unit.

All dendrites and spines within images were traced using Imaris Filament Tracer (RRID: SCR_007366, Bitplane, Switzerland) and analyzed using Imaris software (version 7.2.1, RRID: SCR_007370). Spines were subsequently classified with the following morphological criteria as described previously [43] with a minor modification: mushroom spines were identified as spines with head maximal diameter/neck mean diameter >1.1 and head maximal diameter >0.6 μm; stubby spines were identified as spines with head maximal diameter/neck mean diameter <1.1; spine length/head maximal diameter <2.5; and thin spines were the remaining spines.

### Quantification and statistical analysis

The sample sizes were not pre-determined. For all molecular biology experiments, cellular biology experiments, and behavior tests, sample size was chosen according to previous studies [25, 42, 44]. No animals were excluded from the analyses. Animals were allocated randomly into experimental groups. The investigators were not blinded to group allocation during data collection, since only the investigator who gave handling habituation to mice can access to mice to avoid any types of stress. Also, behavior was recorded by camera to avoid observer bias. Data analysis was performed using Prism 6 Statistics software (RRID: SCR_002798, GraphPad Software, Inc., La Jolla, USA). In all experiments, except for the novel-object recognition test and the passive avoidance tests, one-way analysis of variance (ANOVA) followed by Tukey’s multiple-comparison test was used. Student’s *t* test (two-tailed) was used to compare the differences between two groups. In the novel-object recognition test, repeated two-way ANOVA followed by Tukey’s multiple-comparison test was used to assess significance for experiments with two independent variables. Normal distribution of data was checked by Shapiro–Wilk test. Heteroscedasticity was checked by Brown–Forsythe test (for one-way ANOVA), *F* test (for Student’s *t* test), or Spearman’s test (for two-way ANOVA). If necessary, data sets were square root transformed to enable further statistic testing. In the passive avoidance tests, the Kruskal–Wallis *H* test followed by Dunn’s multiple-comparison test or Mann–Whitney *U* test was used because the distribution was skewed due to the existence of cutoff time.

All data are expressed as the mean ﻿± standard error of the mean of at least three independent experiments. *p* values of <0.05 were considered statistically significant. Detailed information is provided in Table S1.

## Results

### Phosphoproteomics analysis of PKC signaling in striatal/accumbal slices ex vivo

**Fig. 1:**
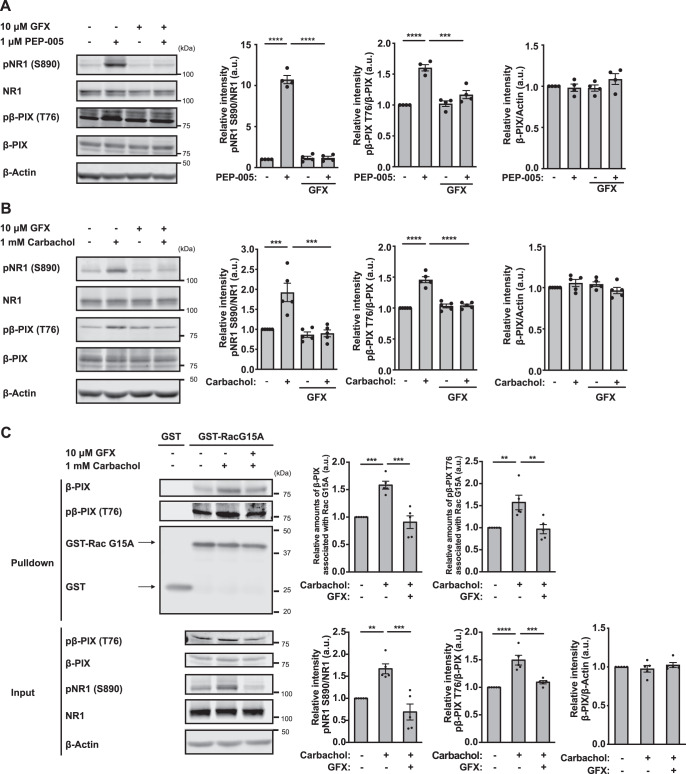
ACh enhanced the Rac-GEF activity of β-PIX through PKC in striatal/accumbal slices ex vivo. **A** Striatal/accumbal slices were pretreated with GF109203X (10 μM, 90 min) at 30 °C and then stimulated with PEP-005 (1 μM, 90 min) at 30 °C. NR1 (S890) and β-PIX phosphorylation (T76) were quantified by immunoblotting over four independent experiments. One-way ANOVA followed by Tukey’s test, ***p < 0.001, ****p < 0.0001. **B** The striatal/accumbal slices were pretreated with GF109203X (10 μM, 90 min) and were then stimulated with carbachol (1 mM, 5 min). NR1 (S890) and β-PIX phosphorylation (T76) were quantified by immunoblotting over five independent experiments. One-way ANOVA followed by Tukey’s test, ***p < 0.001, ****p < 0.001. **C** Rac-GEF activity was analyzed by Rac G15A affinity binding assays. The striatal/accumbal slices were pretreated with the PKC inhibitor GF109203X (10 μM, 90 min) and were then stimulated with carbachol (1 mM, 5 min). The brain slice lysates were subjected to a Rac G15A affinity binding assay. The pulled-down proteins and inputs were immunoblotted with the indicated antibodies (left panels). Quantification of the immunoblotting assay is shown in the right panels. The error bars represent the mean ± SEM of five independent experiments. One-way ANOVA followed by Tukey’s test, **p < 0.01, ***p < 0.001, ****p < 0.0001.

We first examined whether ACh stimulated PKC by monitoring the phosphorylation of the known PKC substrate N-methyl-D-aspartate receptor subunit NR1 [45]. Treatment of striatal/accumbal slices with the PKC activator PEP-005 or ACh receptor agonist carbachol increased the phosphorylation of NR1 at S890 (Fig. 1A, B), whereas pretreatment with the PKC inhibitor GF109203X prevented the phosphorylation of NR1, indicating that ACh activates PKC in the striatum/NAc (Fig. 1A, B).

To screen for novel PKC substrate candidates downstream of ACh, we performed the KIOSS approach using striatal/accumbal slices treated with PEP-005 or carbachol. The cell extracts from striatal/accumbal slices were subjected to affinity beads coated with phospho-Ser/Thr binding protein 14-3-3, which preferentially interacts with signaling molecules and alters the localization, activity, and stability of the target proteins [46, 47], to enrich Ser- or Thr-phosphorylated proteins as described previously (Fig. S1A) [24, 25]. The bound proteins were digested with trypsin and subjected to liquid chromatography-tandem mass spectrometry (LC-MS/MS) to identify the phosphorylated proteins and their phosphorylation sites. The identified proteins were considered PKC substrate candidates if they fulfilled two criteria: (1) the phosphorylation level of the protein based on the ion intensity was increased more than two times by either PEP-005 or carbachol stimulation and (2) the phosphorylation of the protein was decreased by treatment with the PKC inhibitor GF109203X. A total of 116 PKC candidate substrates were identified under PEP-005 and carbachol stimulation (Fig. S1B, C). Detailed information about the phosphorylation sites will be available in the Kinase-Associated Neural Phospho-Signaling database (https://kanphos.neuroinf.jp/dev), which we originally developed [24, 48]. Of note, most of these substrate candidates and phosphorylation sites have not been reported as ACh signals and/or PKC substrates.

### Enhanced Rac-GEF activity of β-PIX by the ACh-PKC cascade in striatal/accumbal slices ex vivo

In silico analysis of the identified PKC substrate candidates using the Reactome database (http://www.reactome.org) revealed that the small GTPase Rac1 pathway, including β-PIX and Bcr, potassium channels, including KCNQ2 and 5, and postsynaptic proteins, including Dlg4 and Shank3, are the major PKC-related pathways. Rac1 signaling is critical for learning and memory because it regulates the structural plasticity of dendritic spines [49], but its linkage with ACh has not been examined.

Among the putative PKC substrates of the Rac1 pathway, β-PIX is a Rac1 guanine nucleotide exchange factor (GEF), and Bcr is a Rac1 GTPase activating protein. We subsequently focused on the Rac1 pathway, especially β-PIX, because GEF is primarily responsible for the activation of Rac1.

We identified the PEP-005-induced phosphorylation site of β-PIX as T76 and prepared an antibody against β-PIX phosphorylated at T76 [50]. By using the phosphorylation-specific antibody, we examined whether carbachol stimulated β-PIX phosphorylation through PKC ex vivo. Treatment of the striatal/accumbal slices with PEP-005 or carbachol increased β-PIX phosphorylation at T76 (Fig. 1A, B). Pretreatment with the PKC inhibitor GF109203X blocked β-PIX phosphorylation induced by PEP-005 or carbachol (Fig. 1A, B). These findings indicate that ACh enhances β-PIX phosphorylation through PKC. Furthermore, carbachol significantly increased β-PIX phosphorylation in a concentration dependent manner starting from 10 μM (Fig. S2), indicating that ACh enhances β-PIX phosphorylation at the physiological ACh concentration.

To further examine whether ACh enhances the Rac-GEF activity of β-PIX through β-PIX phosphorylation by PKC, we treated striatal/accumbal slices with carbachol and performed an affinity precipitation assay using the GST-tagged Rac G15A mutant to specifically pull-down the active form of β-PIX from brain slice lysates [39, 40]. Carbachol treatment increased the amount of total β-PIX and phospho-β-PIX (T76) that were pulled down (Fig. 1C). However, in the presence of the PKC inhibitor GF109203X, carbachol treatment did not increase the amount of total β-PIX and phospho-β-PIX (T76) (Fig. 1C). These results indicate that the ACh-PKC cascade enhances the Rac1-GEF activity of β-PIX.

The above results collectively suggest that ACh activates the Rac1 pathway through β-PIX phosphorylation by PKC. β-PIX is known to interact with PAK and Git1, which was also identified as the putative PKC substrate (Fig. S1C). β-PIX, Git1 and PAK have been implicated in the structural plasticity of dendritic spines [27, 28]. Rac1 is known to activate the PAK-LIMK-cofilin cascade to regulate actin polymerization [51], which led us to investigate whether the ACh-M1R-PKC cascade activates PAK signaling.

### Activation of PAK signaling by the ACh-M1R-PKC cascade in striatum/accumbal slices ex vivo

**Fig. 2:**
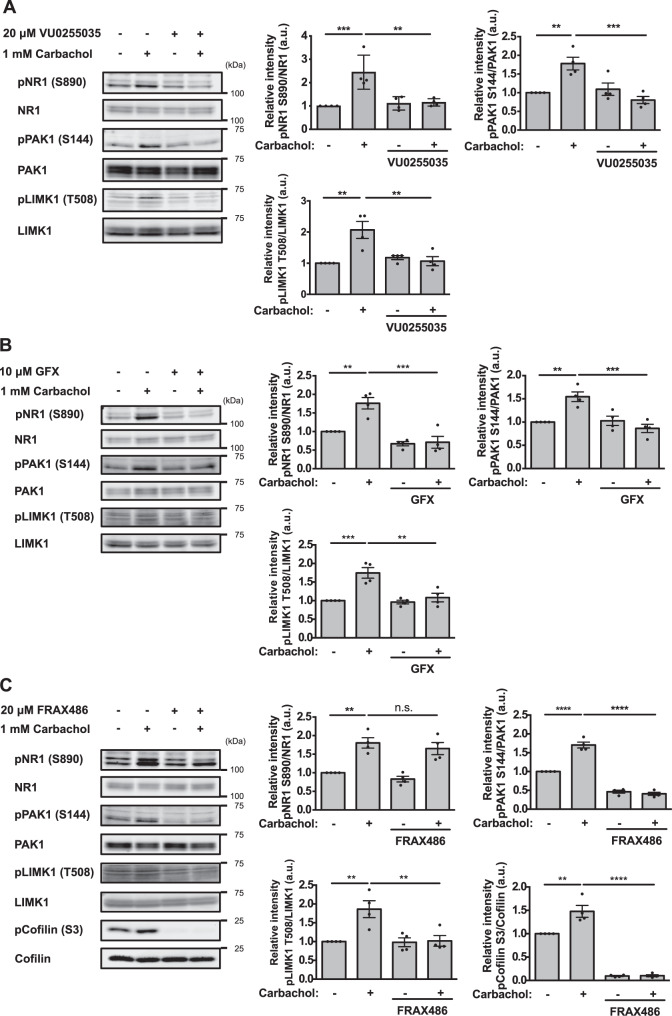
The ACh-M1R-PKC cascade activates PAK signaling in striatum/accumbal slices ex vivo. **A**, **B** Striatal/accumbal slices were pretreated with the M1R-specific antagonist VU0255035 (20 μM) or the PKC inhibitor GF109203X (10 μM) for 90 min and were then stimulated with carbachol for 5 min. NR1 phosphorylation, PAK autophosphorylation, and LIMK phosphorylation were quantified by immunoblotting over four independent experiments. One-way ANOVA followed by Tukey’s test, **p < 0.01, ***p < 0.001. **C** Striatal/accumbal slices were pretreated with the group I-specific PAK inhibitor FRAX486 (20 μM) for 6 h and were then treated with carbachol (1 mM) for 5 min. NR1 phosphorylation, PAK autophosphorylation, LIMK phosphorylation, and cofilin phosphorylation were quantified by immunoblotting over four independent experiments. The error bars represent the mean ± SEM. One-way ANOVA followed by Tukey’s test, n.s. not significant, **p < 0.01, ****p < 0.0001.

To investigate whether ACh activates PAK signaling through M1R, we pretreated striatal/accumbal slices with the M1R-specific antagonist VU0255035 before treatment with carbachol, a nonselective ACh receptor agonist. PAK activation was monitored based on PAK1 autophosphorylation at S144 [pPAK1 (S144)] and the phosphorylation of its main downstream effector LIMK1 at T508 [52, 53]. Carbachol treatment increased PAK1 autophosphorylation and LIMK1 phosphorylation, which were blocked by VU0255035 pretreatment (Fig. 2A). Similarly, pretreatment with the PKC inhibitor GF109203X blocked the enhanced PAK autophosphorylation and LIMK phosphorylation induced by carbachol treatment (Fig. 2B), indicating that ACh activates PAK through the M1R-PKC cascade. We further examined whether ACh activates downstream PAK signaling by monitoring the activation of the PAK-LIMK-cofilin cascade. To this end, striatal/accumbens slices were pretreated with the group I-specific PAK inhibitor FRAX486 before carbachol treatment. FRAX486 pretreatment blocked the enhanced PAK autophosphorylation, LIMK phosphorylation, and cofilin phosphorylation induced by carbachol (Fig. 2C). These findings indicate that ACh activates the PAK-LIMK-cofilin cascade. In addition, carbachol stimulation enhanced PAK autophosphorylation in a concentration dependent manner starting from 10 μM (Fig. S2), indicating that ACh activates PAK at a physiological concentration.

Furthermore, to analyze whether M1R activation itself activates PAK, striatal/accumbal slices were treated with the M1R-specific agonist VU0364572. VU0364572 treatment increased NR1 phosphorylation and PAK autophosphorylation, which were inhibited by pretreatment with the PKC inhibitor GF109203X (Fig. S3A). From these observations, we conclude that ACh-M1R-PKC signaling activates β-PIX-Rac1-PAK-LIMK-cofilin pathway ex vivo.

### Activation of the M1R-PKC-PAK cascade by the cholinesterase inhibitor donepezil in accumbal D2R-MSNs in vivo

**Fig. 3:**
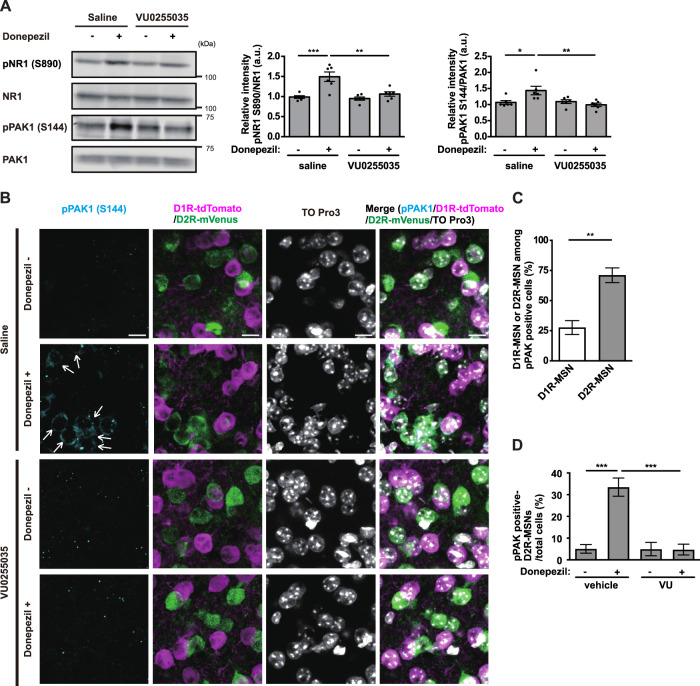
The cholinesterase inhibitor donepezil activates the M1R-PKC-PAK cascade in accumbal D2R-MSNs in vivo. **A** C57BL/6J mice were preadministered the M1R antagonist VU0255035 [40 mg/kg, intraperitoneal (i.p.)] 25 min before donepezil administration [0.4 mg/kg, subcutaneous (s.c.)]. Thirty minutes after donepezil administration, the mice were subjected to immunoblotting analysis. *n* = 6. The error bars represent the mean ± SEM. One-way ANOVA was followed by Tukey’s test, *p < 0.05, **p < 0.01, ***p < 0.001. **B** Drd1a-tdTomato/Drd2-mVenus double transgenic mice were preadministered the M1R antagonist VU0255035 (40 mg/kg, i.p.) 25 min before donepezil administration (0.4 mg/kg, s.c.). Thirty minutes after donepezil administration, the mice were subjected to immunohistochemical analysis. The striatal/accumbal slices (25 μm) were stained with anti-pPAK1 (S144) and anti-GFP antibodies. Nuclei were visualized with TO-PRO3. Arrows indicate pPAK1 (S144)-positive D2R-MSNs. Scale bar, 10 μm. **C** The data plot shows the percentage of D1R-MSNs or D2R-MSNs among pPAK1 (S144)-positive cells when mice were administered with donepezil (0.4 mg/kg, s.c.). The error bars represent the mean ± SEM of three independent experiments. Student’s *t* test, **p < 0.01. **D** Quantification of the pPAK1 (S144)-positive D2R-MSNs in the NAc core. The error bars represent the mean ± SEM of three independent experiments. One-way ANOVA followed by Tukey’s test, ***p < 0.001.

We next examined whether ACh-M1R-PKC signaling activates PAK in the NAc in vivo. To this end, mice were subcutaneously injected with donepezil at the neck scruff to avoid blood circulation, and the NAc was punched out 30 min after the injection. A single dose of donepezil increased NR1 phosphorylation and PAK autophosphorylation, which were inhibited by preadministration of the M1R-specific antagonist VU0255035 (Fig. 3A). Furthermore, a single dose of the M1R-specific agonist VU0364572 by intraperitoneal injection increased NR1 phosphorylation and PAK autophosphorylation (Fig. S3B). Based on the finding that ACh-PKC cascade enhances the Rac1-GEF activity of β-PIX (Fig. 1C), we further examined the involvement of β-PIX phosphorylation by PKC in response to donepezil administration. To this end, mice were injected with PKC-specific inhibitor NPC-15437 [54], which has been shown to prevent aversive learning [55]. Donepezil administration increased NR1 phosphorylation, β-PIX phosphorylation and PAK autophosphorylation, which were inhibited by preadministration of NPC-15437 (Fig. S4A). Collectively, these results indicate that ACh-M1R-PKC-β-PIX signaling activates PAK in the NAc in vivo.

Because the activation of D2R-MSNs is critical in aversive learning, we assessed PAK activation in accumbal D2R-MSNs by donepezil injection. To this end, *Drd1a-tdTomato*/*Drd2-mVenus* double transgenic mice, in which the D1R-MSNs express tandem dimeric red fluorescent protein (tdTomato) and the D2R-MSNs express monomeric yellow fluorescent protein (mVenus), were subjected to immunohistochemical analysis in the NAc core. Donepezil injection increased the number of pPAK1 (S144)-positive cells (Fig. 3B, D). Among them, ~70% were D2R-MSNs (Fig. 3C). Preadministration of the M1R-specific antagonist VU0255035 reduced the number of pPAK1 (S144)-positive D2R-MSNs (Fig. 3B, D), indicating that ACh activates PAK through M1R in accumbal D2R-MSNs in vivo.

### Involvement of M1R-PKC-PAK-β-PIX cascade in aversive learning

**Fig. 4:**
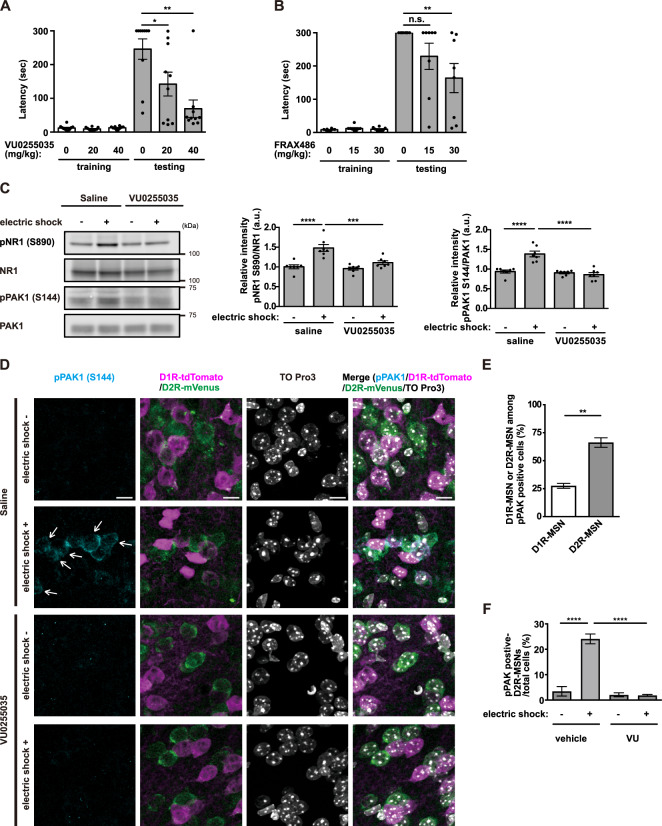
Electric foot shock activates PAK through M1Rs in accumbal D2R-MSNs. **A** VU0255035 was administered i.p. 25 min before the electric foot shock (0.4 mA, 2.0 s). *n* = 10. Error bars indicate mean ± SEM. Kruskal–Wallis test, followed by Dunn’s multiple comparisons test, *p < 0.05, **p < 0.01. **B** FRAX486 was administered s.c. 6 h before the electric foot shock (0.4 mA, 2.0 s). *n* = 8. Error bars indicate mean ± SEM. Kruskal–Wallis test, followed by Dunn’s multiple comparisons test, n.s. not significant, **p < 0.01. **C** VU0255035 was administered (40 mg/kg, i.p.) before the electric foot shock (0.4 mA, 2.0 s). Ten minutes after electric foot shock, NR1 phosphorylation and PAK autophosphorylation were quantified by immunoblotting. *n* = 7. Error bars indicate mean ± SEM. One-way ANOVA followed by Tukey’s test, ***p < 0.001, ****p < 0.0001. **D** Drd1a-tdTomato/Drd2-mVenus double transgenic mice were administered with VU0255035 (40 mg/kg, i.p.) before the electric foot shock. Ten minutes after electric foot shock, immunohistochemical analysis was performed. Immunofluorescence staining using an anti-pPAK1 (S144) antibody, an anti-GFP antibody, and TO-PRO3 is shown. Arrows indicate pPAK1 (S144)-positive D2R-MSNs. Scale bar, 10 μm. **E** The percentage of D1R-MSNs or D2R-MSNs among pPAK1 (S144)-positive cells after electric foot shock. Error bars indicate the mean ± SEM of three independent experiments. Student’s *t* test, **p < 0.01. **F** Quantification of the pPAK1 (S144)-positive D2R-MSNs in the NAc core. Error bars indicate the mean ± SEM of three independent experiments. One-way ANOVA followed by Tukey’s test, ****p < 0.0001.

We next performed the passive avoidance test using an electric foot shock (0.4 mA, 2.0 s) to examine whether M1R and PAK activation are involved in aversive learning. A single dose of the M1R-specific antagonist VU0255035 significantly decreased the step-through latency in a dose-dependent manner (Fig. 4A). Similarly, a single dose of the PAK-specific antagonist FRAX486 significantly decreased the step-through latency in a dose-dependent manner (Fig. 4B). These results suggest that M1R and PAK activation are involved in aversive learning.

To examine whether electric foot shock activates M1R-PKC-PAK signaling in the NAc, we trained mice in the step-through passive avoidance procedure mentioned above. Ten minutes after the electric foot shock (0.4 mA, 2.0 s), the NAc was punched out, and NR1 phosphorylation and PAK autophosphorylation were monitored by immunoblot analysis. Both NR1 phosphorylation and PAK autophosphorylation were significantly elevated after the electric foot shock and were inhibited by preadministration of the M1R-specific antagonist VU02550355 (Fig. 4C), indicating that M1R is involved in electric foot shock-induced PAK autophosphorylation. Also, preadministration of PKC-specific inhibitor NPC- 15437 inhibited the enhanced NR1 phosphorylation, β-PIX phosphorylation, and PAK autophosphorylation induced by electric foot shock (Fig. S4B), indicating that PKC is involved in electric foot shock-induced β-PIX phosphorylation and PAK autophosphorylation. These findings indicate that electric foot shock activates PAK signaling through the M1R-PKC cascade in the NAc.

For further assessment of PAK activation in accumbal D2R-MSNs by electric foot shock, *Drd1a-tdTomato*/*Drd2-mVenus* double transgenic mice were subjected to immunohistochemical analysis, and the number of pPAK1 (S144)-positive D2R-MSNs in the NAc core was determined. Immunohistochemical analysis revealed that the electric foot shock (0.4 mA, 2.0 s) increased the number of pPAK1 (S144)-positive cells (Fig. 4D, F). Among them, ~70% were D2R-MSNs (Fig. 4E). Preadministration of the M1R-specific inhibitor VU0255035 reduced the number of pPAK1 (S144)-positive D2R-MSNs (Fig. 4D, F), indicating that M1R is involved in electric foot shock-induced PAK autophosphorylation in D2R-MSNs. We further examined if β-PIX is phosphorylated by PKC in D2R-MSNs in response to electric foot shock. Immunohistochemical analysis revealed that the electric foot shock (0.4 mA, 2.0 s) increased the number of pβ-PIX (T76)-positive cells (Fig. S5A, C). Among them, ~70% were D2R-MSNs (Fig. S5B). Preadministration of the PKC-specific inhibitor NPC 15437 reduced the number of pβ-PIX (T76)-positive D2R-MSNs (Fig. S5A, C), indicating that PKC is involved in electric foot shock-induced β-PIX phosphorylation in D2R-MSNs. The above findings (Fig. 4F and Figs. S4B and S5C) collectively suggest that M1R-PKC-β-PIX-PAK cascade in D2R-MSN is involved in aversive learning.

**Fig. 5:**
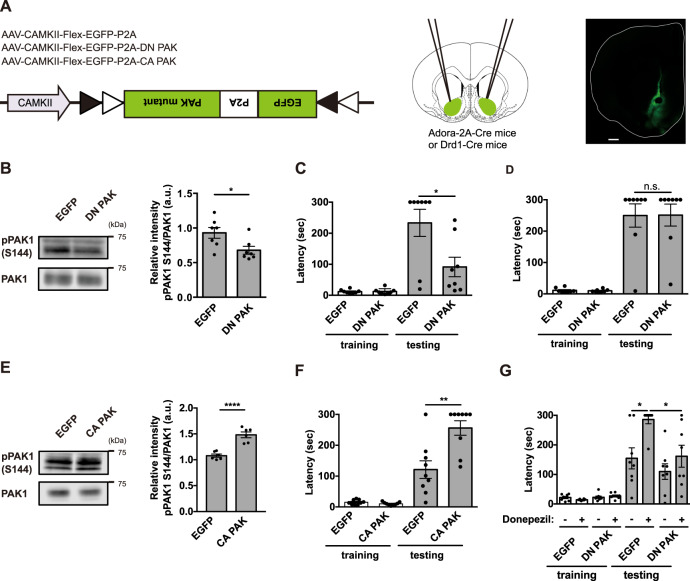
Donepezil enhances aversive learning through PAK activation in accumbal D2R-MSNs. **A** Schematic of AAV-mediated PAK expression in accumbal D2R-MSNs or D1R-MSNs. Left panel: AAV constructs. Middle panel: stereotaxic injection of AAVs into the NAc of Adora-2a-Cre or Drd1-Cre transgenic mice. Right panel: representative coronal brain slice expressing EGFP-P2A-CA PAK. Scale bar, 500 μm. **B** AAV-mediated expression of DN PAK in D2R-MSNs inhibits PAK autophosphorylation in the NAc. Left panels: representative immunoblots. Right panel: quantification of the immunoblotting assay. Error bars indicate mean ± SEM. *n* = 7. Student’s *t* test, *p < 0.05. **C** AAV-mediated expression of DN PAK in D2R-MSNs in Adora-2a-Cre transgenic mice decreased the step-through latency. Error bars indicate mean ± SEM. *n* = 8. Mann–Whitney *U* test, *p < 0.05. **D** AAV-mediated expression of DN PAK in D1R-MSNs in Drd1-Cre transgenic mice did not alter the step-through latency. Error bars indicate mean ± SEM. *n* = 8. Mann–Whitney *U* test, n.s. not significant. **E** AAV-mediated expression of CA PAK in D2R-MSNs increased PAK autophosphorylation in the NAc. Left panels: representative immunoblots. Right panel: quantification of the immunoblotting assay. Error bars indicate mean ± SEM. *n* = 6. Student’s *t* test., ****p < 0.0001. **F** AAV-mediated expression of CA PAK in D2R-MSNs in Adora-2a-Cre transgenic mice increased the step-through latency. Error bars indicate mean ± SEM. *n* = 9. Mann–Whitney *U* test, **p < 0.01. **G** Donepezil was administered (0.4 mg/kg, s.c.) 30 min before the electric foot shock (0.3 mA, 1.0 s). *n* = 8. Kruskal–Wallis *H* test, followed by Dunn’s multiple comparisons test, *p < 0.05.

To investigate whether PAK activity in D2R-MSNs is involved in aversive learning, we used the Cre-Flex system to conditionally express a DN PAK mutant [autoinhibitory domain of PAK (83 aa–149 aa)] that inhibits endogenous group I PAK activity in D2R-MSNs. In this system, Flex-AAVs encoding the DN PAK mutant were injected into the NAc of Adora-2a-Cre transgenic mice [56, 57], in which Cre recombinase inverts the DN PAK gene to sense orientation specifically in D2R-MSNs (Fig. 5A). To confirm that the DN PAK mutant inhibits the autophosphorylation level of PAK in the NAc in vivo, Adora-2A-Cre transgenic mice expressing the DN PAK mutant were subjected to immunoblot analysis 3 weeks after Flex-AAV injection. Immunoblot analysis revealed that the expression of DN PAK decreased PAK1 autophosphorylation at S144 compared with the expression of EGFP (Fig. 5B). To investigate the involvement of the DN PAK mutant in aversive learning, Adora-2a-Cre transgenic mice and Drd1-Cre transgenic mice were injected with Flex-AAV encoding DN PAK into the NAc and were then subjected to a passive avoidance test 3 weeks later. The results showed that the DN PAK mutant in the accumbal D2R-MSNs (Fig. 5C), but not in the accumbal D1R-MSNs (Fig. 5D), significantly decreased the step-through latency 24 h after electric foot shock (0.4 mA, 2.0 s), indicating that PAK activation specifically in the accumbal D2R-MSNs is required for aversive learning. Based on the finding that the Rac-GEF activity of β-PIX was enhanced by the ACh-PKC cascade (Fig. 1C), we investigated whether Rac1 activation in D2R-MSNs is involved in aversive learning. The expression of DN Rac1 in the D2R-MSNs significantly reduced the step-through latency (Fig. S6), indicating that the Rac1-PAK cascade in the accumbal D2R-MSNs is involved in aversive learning.

To further investigate whether PAK activation in D2R-MSNs is sufficient for enhanced aversive learning, we injected Flex-AAV expressing the CA PAK mutant [catalytic domain of PAK1 (248 aa–543 aa)] into the NAc of Adora-2A-Cre transgenic mice (Fig. 5A). Three weeks after the injection, an immunoblotting analysis revealed that the expression of CA PAK increased PAK autophosphorylation in the NAc in vivo (Fig. 5E). To test whether the expression of CA PAK in accumbal D2R-MSNs potentiates aversive learning, we subjected Adora-2A-Cre transgenic mice expressing CA PAK to a passive avoidance test in which mild electric foot shock (0.3 mA, 1.0 s) was used as an aversive stimulus. The expression of the CA PAK mutant in the accumbal D2R-MSNs significantly increased the step-through latency 24 h after electric foot shock (Fig. 5F), indicating that PAK activation in the accumbal D2R-MSNs enhances aversive learning.

### Involvement of PAK in the structural plasticity of dendritic spines of accumbal D2R-MSNs

Because group I PAKs and their downstream effector LIMK1-cofilin have been implicated in regulating learning and memory through spine structural plasticity of hippocampal and cortical pyramidal neurons [58, 59], we examined the effect of PAK mutants on the spine structural plasticity of accumbal D2R-MSNs in vivo. To this end, Adora-2a-Cre mice were injected with Flex-AAV encoding DN PAK or CA PAK and were then subjected to spine analysis. The results showed that the expression of the DN PAK mutant in the accumbal D2R-MSNs decreased the total spine density, thin spine density, and spine length, whereas the expression of CA PAK increased the total spine density, mushroom spine density, spine volume, and spine head diameter (Fig. S7A–F), indicating that PAK activity is involved in the structural synaptic plasticity of accumbal D2R-MSNs in vivo.

### Enhanced aversive learning by donepezil through PAK activation in accumbal D2R-MSNs

Based on the finding that donepezil activated PAK through M1R in accumbal D2R-MSNs in Fig. 3B, we examined whether donepezil enhances aversive learning by performing a passive avoidance test using a mild electric foot shock (0.3 mA, 1.0 s). Adora-2A-Cre mice expressing either EGFP or the DN PAK mutant in the NAc were subcutaneously injected with donepezil and subjected to the passive avoidance test to assess this hypothesis. Donepezil injection itself significantly increased the step-through latency 24 h after electric foot shock (Fig. 5G). Furthermore, DN PAK expression in the accumbal D2R-MSNs attenuated the enhanced step-through latency induced by donepezil injection (Fig. 5G). From the above observations, we conclude that donepezil enhances aversive learning through PAK in accumbal D2R-MSNs.

### PAK activation by donepezil in the hippocampus in vivo

Because the ACh level in the brains of AD patients is decreased not only in the NAc [60] but also in other brain regions that regulate learning and memory, including the hippocampus [1, 2], we tested whether donepezil activates PAK in the hippocampus. To this end, C57BL/6 mice were subcutaneously injected with donepezil at the neck scruff and the dorsal region of hippocampi were punched out 30 min after injection for immunoblotting analysis. Donepezil administration increased PAK autophosphorylation, which was inhibited by the preadministration of PAK inhibitor FRAX486 (Fig. S8A). Similarly, immunohistochemical analysis revealed that preadminsitration of FRAX 486 inhibited the enhanced pPAK1 (S144) staining induced by donepezil administration in the CA1 region of the dorsal hippocampus (Fig. S8B, C). These findings suggest that the identified ACh-PAK cascade can also be applied to hippocampus-dependent learning and memory. To investigate the effect of the ACh-PAK cascade on hippocampus-dependent learning and memory, we performed contextual fear conditioning test, novel object recognition test and Y-maze test by injecting FRAX 486 or donepezil into wild type C57BL/6J mice. FRAX 486 administration into wild type C57BL/6J mice significantly decreased exploratory preference in novel object recognition test (Fig. S9A) and produced a freezing response in the contextual fear conditioning test (Fig. S9B). However, it did not affect spontaneous alternation behavior in Y-maze test (Fig. S9C). These results indicate that PAK is involved in recognition memory and associative learning, but not in short-term memory. On the other hand, donepezil administration into wild type C57BL/6J mice did not affect visual recognition memory in the novel object recognition test (Fig. S9A), associative learning in the contextual fear conditioning test (Fig. S9B), or short-term memory in the Y-maze test (Fig. S9C).

## Discussion

ACh was the first substance proposed to act as a neuromodulator [61]. The isolation of synaptic vesicles from the brain [62] provided a new foundation for the chemical synaptic transmission hypothesis, a concept currently accepted for the brain machinery in learning through the transmission of neuromodulators from one neuron to other neurons. Nevertheless, as stated in the introduction, ACh intracellular signaling beyond M1R in the brain, including accumbal D2R-MSNs that enhance aversive learning, has remained unelucidated for more than 45 years. The present findings together with previous findings indicate that the identified ACh-PKC-β-PIX-Rac1-PAK cascade in accumbal D2R-MSNs enhances aversive learning. The cascade further implies that it enhances aversive learning by regulating the structural synaptic plasticity of D2R-MSNs (Fig. S10).

### The role of PKC signaling in the striatum/NAc

Our phosphoproteomics analysis revealed 116 PKC substrate candidates. Here, we particularly focused on β-PIX and showed that ACh activates PAK by enhancing the Rac-GEF activity of β-PIX (Fig. 1A–C). Several potassium voltage-gated channel proteins, including Kcnh2, Kcnh7, Kcnq2, and Kcnq5, were also identified in the analysis (Fig. S1C). They are postsynaptic proteins known to regulate neuronal excitability. M1R signaling has been implicated to suppress M-current mediated by potassium voltage-gated channel KCNQ2 via PKC signaling activation to modulate neuronal excitability [63, 64]. Also, ACh has been reported to enhance the neuronal excitability of D2R-MSNs [18]. During the revision of this manuscript, we have shown that muscarinic signaling regulates KCNQ2 phosphorylation in the NAc via PKC for aversive learning induced by electric foot shock [65]. Given that KCNQ2 phosphorylation is associated with the modulation of KCNQ2 channel activity [64, 66], it is plausible that neural firing in D2R-MSNs is altered upon aversive stimuli. Further investigation on the involvement of neural firing in D2R-MSNs upon aversive stimuli will be done in the future.

Phosphoproteomics analysis also identified several postsynaptic density proteins as PKC substrate candidates, including Dlg4, Dlgap1, Dlgap2, Dlgap3 and Shank3 (Fig. S1C). They are postsynaptic scaffold proteins localized in the postsynaptic density and are required for synaptic plasticity associated with NMDA receptor signaling [67]. The presynaptic proteins Bassoon, Piccolo, and Rims1 have also been identified as PKC substrate candidates (Fig. S1C). They are scaffold proteins that anchor neurotransmitter-filled synaptic vesicles to the active zone of nerve terminals [68]. Future research on these PKC substrate candidates could identify novel mechanisms involved in the functions not only of the striatum/NAc but also of other brain areas.

### The critical role of ACh-PKC-PAK signaling in D2R-MSN-mediated aversive learning

ACh in the NAc/striatum was shown to preferentially enhance D2R-MSN excitability through M1R [18]. The identification of ACh-PKC-PAK intracellular signaling in D2R-MSNs provides direct evidence that ACh stimulates aversive learning through accumbal D2R-MSNs.

Group I PAKs, PAK1 and PAK3, have been implicated in the structural plasticity of hippocampal and cortical neurons [58], which was confirmed by our present findings that PAK activity contributes to the structural plasticity of dendritic spines in accumbal D2R-MSNs (Fig. S7A–F). In addition to group I PAKs regulating spine structural plasticity through LIMK-cofilin cascade-mediated actin dynamics [58], they regulate other factors implicated in synaptic plasticity, such as transcriptional activity, cell-cell adhesion, cell surface expression of glutamate receptor AMPAR, and mRNA trafficking [69–71]. Future analysis of the PAK substrates downstream of ACh will be beneficial to further understand the molecular basis of aversive learning. Moreover, these studies might reveal another role of ACh in synaptic plasticity, in addition to the prevailing view that ACh regulates MSN excitability [72].

### The intracellular mechanism of AD therapeutic drugs and their effects on learning and memory

Donepezil increases ACh levels in various brain regions by inhibiting cholinesterase and is a major drug for treating memory loss and learning disabilities in AD. The clarification of its intracellular mechanism in this study further strengthens our finding that the ACh-PAK cascade enhances aversive learning through accumbal D2R-MSNs.

Donepezil is also known to enhance cognition involving the hippocampus in AD patients [4]. The present study showed that donepezil activates PAK in the dorsal hippocampus in vivo (Fig. S8A, C). Furthermore, administration of PAK inhibitor FRAX 486 into wild type C57BL/6J mice significantly decreased exploratory preference in the novel object recognition test (Fig. S9A) and decreased the freezing response in the contextual fear conditioning test (Fig. S9B), which indicates that PAK is involved in recognition memory and associative learning. On the other hand, donepezil administration into wild type C57BL/6J mice did not alter recognition memory and associative learning (Fig. S9A, B). Given that M1R is a key mediator of learning and memory, it is plausible that donepezil has a weak effect on M1Rs in hippocampus-dependent learning and memory as the elevated ACh level induced by donepezil activates multiple ACh receptors. As supporting evidence, a recent paper has shown that administration of the M1R-specific agonist HTL9936 into wild type Wistar rats, but not administration of donepezil, enhanced recognition memory in novel object recognition test [73], which is consistent with our finding. Collectively, the above findings imply that the identified ACh-M1R-PAK cascade is also involved in hippocampus-dependent learning and memory.

### Outlook—door to new therapeutic strategies for CNS disorders

As shown by the effects of donepezil, ACh is a therapeutic target for treating the AD symptoms of memory loss and learning disabilities. However, the ACh level increase by donepezil causes various side effects due to the activation of multiple ACh receptors. Recent findings have shown that administration of a M1R-specific agonist enhances learning and memory tasks, which emphasizes the importance of targeting the M1R pathway [73]. In this study, we identified the downstream signaling pathway beyond the ACh receptor linked with learning and memory.

The present study provides evidence that donepezil activates ACh-M1R-PKC-Rac1-PAK intracellular signaling in accumbal D2R-MSNs, thereby stimulating aversive learning. Furthermore, it provides evidence that the ACh-PAK cascade is involved in recognition memory and associative learning. Recently, PAK-mediated pathways have been considered potential therapeutic targets for various CNS disorders based on the involvement of PAK dysfunction in pathogenesis [74–76]. Based on the fact that aversive learning, recognition memory, and associative learning are used as outputs for AD drug screening [73], our findings imply that the ACh-PAK cascade is a potential therapeutic target for AD. Future studies using AD animal models are required to warrant translation of this approach.

## Supplementary information

Supplementary figure legends

Supplementary Figure 1

Supplementary Figure 2

Supplementary Figure 3

Supplementary Figure 4

Supplementary Figure 5

Supplementary figure 6

Supplementary Figure 7

Supplementary Figure 8

Supplementary Figure 9

Supplementary Figure 10

Supplementary table 1
