# Supplementary material for: Phosphoproteomic of the acetylcholine pathway enables discovery of the PKC-β-PIX-Rac1-PAK cascade as a stimulatory signal for aversive learning
Source: Mol Psychiatry. 2022 Jun 3;27(8):3479–92. doi: 10.1038/s41380-022-01643-2 (PMC9708603; doi:10.1038/s41380-022-01643-2)
Supplement: Supplementary file 1 — Supplementary figure legends [file 41380_2022_1643_MOESM1_ESM.docx]

**Fig. S1 Phosphoproteomics analysis revealed 116 PKC substrate candidates in striatal/accumbal slices *ex vivo*.** **(A)** Scheme of the KIOSS method used to identify PKC substrate candidates downstream of ACh. Striatal/accumbal slices were treated with the acetylcholine agonist carbachol (1 mM, 5 min) or the PKC activator PEP-005 (1 μM, 90 min) to induce PKC-mediated phosphorylation. Extracts of these striatal/accumbal slices were then applied to affinity beads coated with GST-tagged 14-3-3 to pull down phosphorylated proteins. The bound proteins were subjected to LC-MS/MS to identify the phosphorylated proteins and their phosphorylation sites. **(B)** Venn diagrams showing the number of identified phosphoproteins. **(C)** A total of 116 proteins were identified under PEP-005 and carbachol stimulation.

**Fig. S2 Acetylcholine agonist carbachol enhanced NR1 phosphorylation, β-PIX phosphorylation, and PAK autophosphorylation in a concentration dependent manner in striatal/accumbal slices.** Striatal/accumbal slices were treated with the acetylcholine agonist carbachol for 5 min. NR1 phosphorylation, β-PIX phosphorylation, and PAK autophosphorylation were quantified by immunoblotting over five independent experiments. One-way ANOVA followed by Tukey’s test, * p < 0.05, ** p < 0.01, *** p < 0.001, **** p < 0.0001.

**Fig. S3 The M1R agonist VU0364572 increased PAK** **autophosphorylation in the striatum/NAc both *ex vivo* and *in vivo*. (A)** Striatal/accumbal slices were pretreated with the PKC inhibitor GF109203X (10 μM) for 90 min and were then stimulated with the M1R-specific agonist VU0364572 (30 μM) for 25 min. NR1 phosphorylation and PAK autophosphorylation were quantified by immunoblotting over five independent experiments. One-way ANOVA followed by Tukey’s test, * p < 0.05, ** p < 0.01. **(B)** C57BL/6J mice were administered with the M1R agonist VU0364572 (25 mg/kg, i.p.). Thirty minutes later, the mice were subjected to immunoblotting analysis. n=8. The error bars represent the mean ± SEM. Student’s *t* test, ** p < 0.01, **** p < 0.0001.

**Fig. S4 Donepezil or electric foot shock enhances β-PIX phosphorylation and PAK autophosphorylation through PKC in NAc *in vivo*. (A)**C57BL/6J mice were administered the PKC antagonist NPC 15437 [1 mg/kg, intraperitoneal (s.c.)] 30 min before donepezil administration [0.4 mg/kg, subcutaneous (s.c.)]. Thirty minutes after donepezil administration, the mice tissues were subjected to immunoblotting analysis. n=9. The error bars represent the mean ± SEM. One-way ANOVA was followed by Tukey’s test, ** p < 0.01, *** p < 0.001, **** p < 0.0001. **(B)** C57BL/6J mice were administered the PKC antagonist NPC 15437 [1 mg/kg, intraperitoneal (s.c.)] 30 min before electric foot shock (0.4 mA, 2 sec). Ten minutes after donepezil administration, the mice tissues were subjected to immunoblotting analysis. n=8. The error bars represent the mean ± SEM. One-way ANOVA was followed by Tukey’s test, ** p < 0.01, *** p < 0.001, **** p < 0.0001.

**Fig. S5 Electric foot shock enhances β-PIX phosphorylation through PKC in accumbal D2R-MSNs. (A)** Drd1a-tdTomato/Drd2-mVenus double transgenic mice were administered NPC15437 (1 mg/kg, i.p.) before the electric foot shock. Ten minutes after electric foot shock, immunohistochemical analysis was performed. Immunofluorescence staining results using an anti-pβ-PIX (T76) antibody, an anti-GFP antibody, and TO-PRO3 are shown. Arrows indicate pβ-PIX (T76)-positive D2R-MSNs. Scale bar, 10 μm. **(B)** The percentage of D1R-MSNs or D2R-MSNs among pβ-PIX (T76)-positive cells after electric foot shock. Error bars indicate the mean ± SEM of three independent experiments. Student’s *t* test, *** p < 0.001. **(C)** Quantification of the pβ-PIX (T76)-positive D2R-MSNs in the NAc core. Error bars indicate the mean ± SEM of three independent experiments. One-way ANOVA followed by Tukey’s test, **** p < 0.0001.

**Fig. S6 Rac activation in D2R-MSNs is involved in aversive learning.** Adora-2a-Cre transgenic mice were injected with Flex-DN Rac AAV. Three weeks after the injection, the mice were subjected to a passive avoidance test. DN Rac in the D2R-MSNs significantly reduced the step-through latency 24 h after electric shock (0.4 mA, 2 sec). The error bars indicate the mean ± SEM. EGFP=9, DN Rac=8. Mann-U Whitney test, ** p<0.01.

**Fig. S7 PAK activity contributes to spine morphogenesis in D2R-MSNs *in vivo*. (A)** Adora-2a-Cre transgenic mice expressing DN PAK in D2R-MSNs were subjected to spine analysis. Striatal/accumbal slices (100 μm) were incubated with GFP antibody. Scale bar, 2 μm. **(B)** Quantification of spine analysis. EGFP: n = 3 mice; DN PAK: n = 3 mice. Error bars indicate mean ± SEM. Student’s *t* test, n.s.: not significant, * p < 0.05. **(C)** Statistical analysis of spine volume, spine head diameter, and spine length. EGFP: n = 3 mice; DN PAK: n = 3 mice. The error bars indicate the mean ± SEM. Student’s *t* test, n.s.: not significant, * p < 0.05. **(D)** Adora-2a-Cre transgenic mice expressing CA PAK were subjected to spine analysis. Striatal/accumbal slices (100 μm) were incubated with GFP antibody. Scale bar, 2 μm. **(E)** Quantification of spine analysis. EGFP: n = 3 mice; CA PAK: n = 3 mice. Error bars indicate mean ± SEM. Student’s *t* test, n.s.: not significant, * p < 0.05. **(F)** Statistical analysis of spine volume, spine head diameter, and spine length. EGFP: n = 3 mice; CA PAK: n = 3 mice. Error bars indicate mean ± SEM. Student’s *t* test, n.s.: not significant, * p < 0.05.

**Fig. S8 Donepezil enhances PAK autophosphorylation in the CA1 hippocampus *in vivo.* (A)** C57BL/6J mice were administered with donepezil (0.4 mg/kg, s.c.). Thirty minutes later, the mice tissues were subjected to immunoblotting analysis. n=9. The error bars represent the mean ± SEM. One-way ANOVA was followed by Tukey’s test, **** p < 0.0001. **(B)** The diagram illustrates cornu ammonis area 1 (CA1), cornu ammonis area 3 (CA3), and dentate gyrus (DG) regions in the dorsal hippocampus. The boxed area indicates the region of interest (ROI). **(C)** C57BL/6J mice were administered with donepezil (0.4 mg/kg, s.c.). Thirty minutes later, the mice were subjected to immunohistochemical analysis. Dorsal hippocampal slices (25 μm) were incubated with the indicated antibodies. Nuclei were visualized with DAPI. Scale bar, 10 μm.

**Fig. S9 PAK is involved in recognition memory and associative learning, but not in short-term memory.** (A) C57BL/6J mice were subjected to the training session in the novel object recognition test 6 hours and 30 minutes after the administration of FRAX486 (40 mg/kg, s.c.) and donepezil (0.4 mg/kg, s.c.), respectively. The retention session was carried out 24 hours after the training session. Exploratory preference during a 10-min session was measured. Error bars indicate mean ± SEM. Repeated two-way ANOVA followed by Tukey’s test, ****p< 0.0001 vs control mice, #### p< 0.0001 vs training session. (B) C57BL/6J mice were subjected to the conditioning phase in the contextual fear conditioned learning test 6 hours and 30 minutes after the administration of FRAX486 (40 mg/kg, s.c.) and donepezil (0.4 mg/kg, s.c.), respectively. The test phase was carried out 24 hours after the conditioning phase. Freezing response during a 2-min phase was measured. Error bars indicate mean ± SEM. One-way ANOVA followed by Tukey’s test, n.s.: not significant, **p< 0.01 vs control mice. (C) C57BL/6J mice were subjected to the Y-maze test 6 hours and 30 minutes after the administration of FRAX486 (40 mg/kg, s.c.) and donepezil (0.4 mg/kg, s.c.), respectively. Alternation behavior during an 8-min session was measured. Error bars indicate mean ± SEM. One-way ANOVA followed by Tukey’s test , n.s.: not significant.

**Fig. S10 Proposed ACh intracellular signaling in accumbal D2R-MSNs that leads to enhanced aversive learning.** In response to aversive stimulation (electric footshock), ACh released from cholinergic interneurons activates PAK through the M1R-PKC-β-PIX cascade in accumbal D2R-MSNs to enhance aversive learning. The cholinesterase inhibitor donepezil acts through the identified ACh-PAK pathway in accumbal D2R-MSNs to promote aversive learning.
