# Supplementary figures and images for: Phosphoproteomic of the acetylcholine pathway enables discovery of the PKC-β-PIX-Rac1-PAK cascade as a stimulatory signal for aversive learning
Source: Mol Psychiatry. 2022 Jun 3;27(8):3479–92. doi: 10.1038/s41380-022-01643-2 (PMC9708603; doi:10.1038/s41380-022-01643-2)

Figure S1

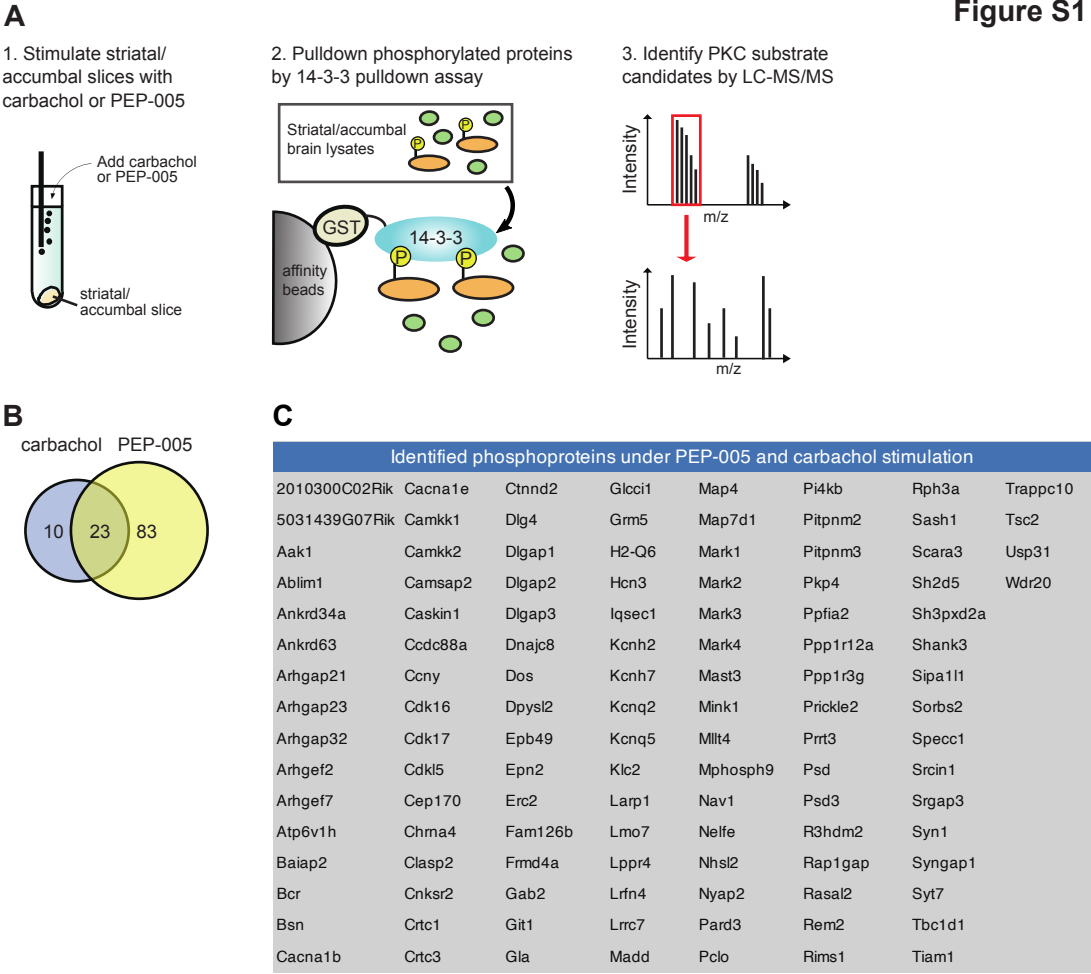

Supplement: Supplementary file 2 — Supplementary Figure 1 [file 41380_2022_1643_MOESM2_ESM.pdf]

**Figure S2**

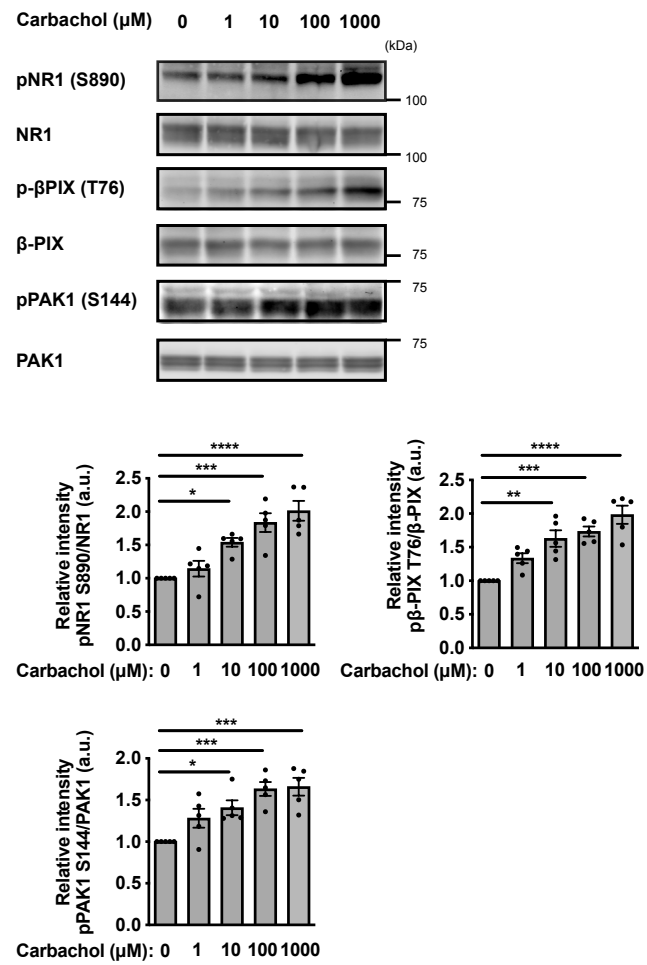

Supplement: Supplementary file 3 — Supplementary Figure 2 [file 41380_2022_1643_MOESM3_ESM.pdf]

**Figure S3**

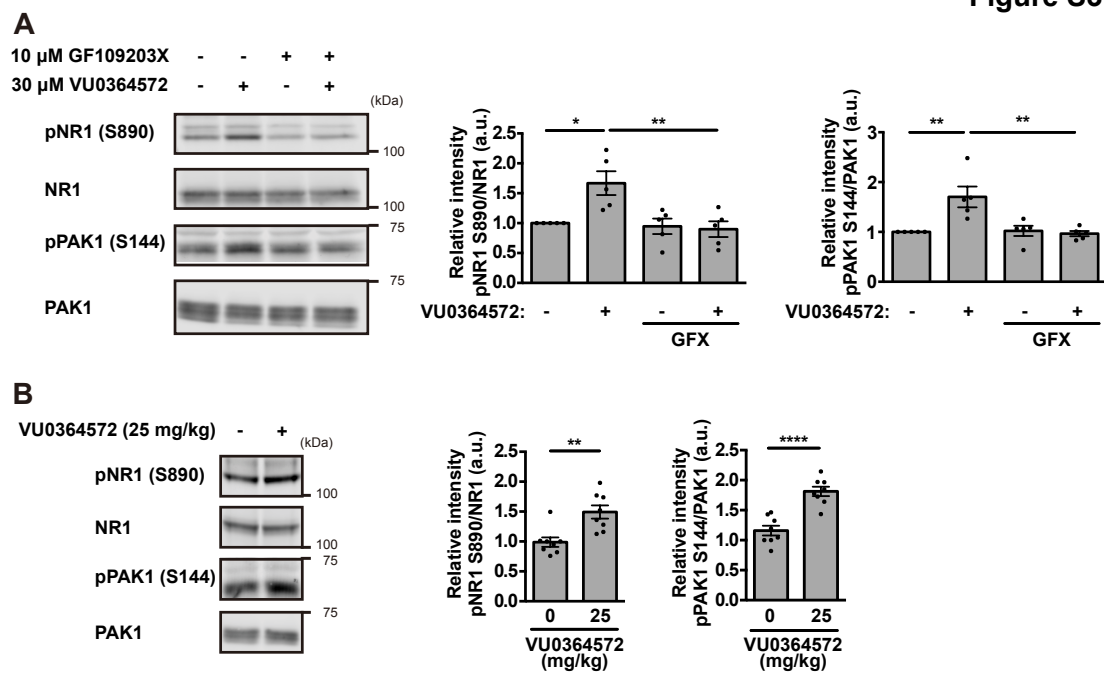

Supplement: Supplementary file 4 — Supplementary Figure 3 [file 41380_2022_1643_MOESM4_ESM.pdf]

Figure S4

**A**

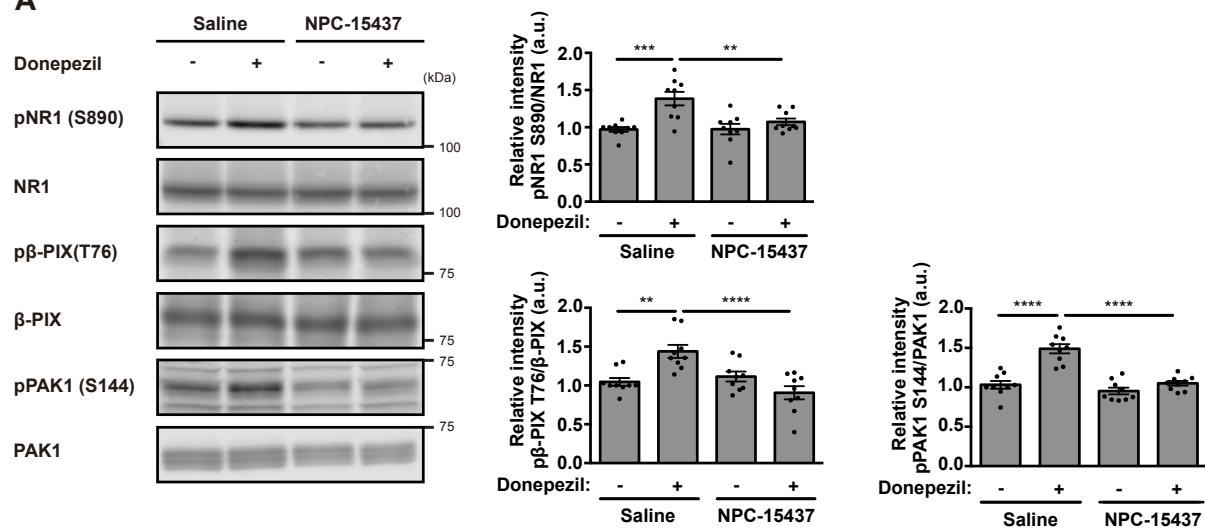

**B**

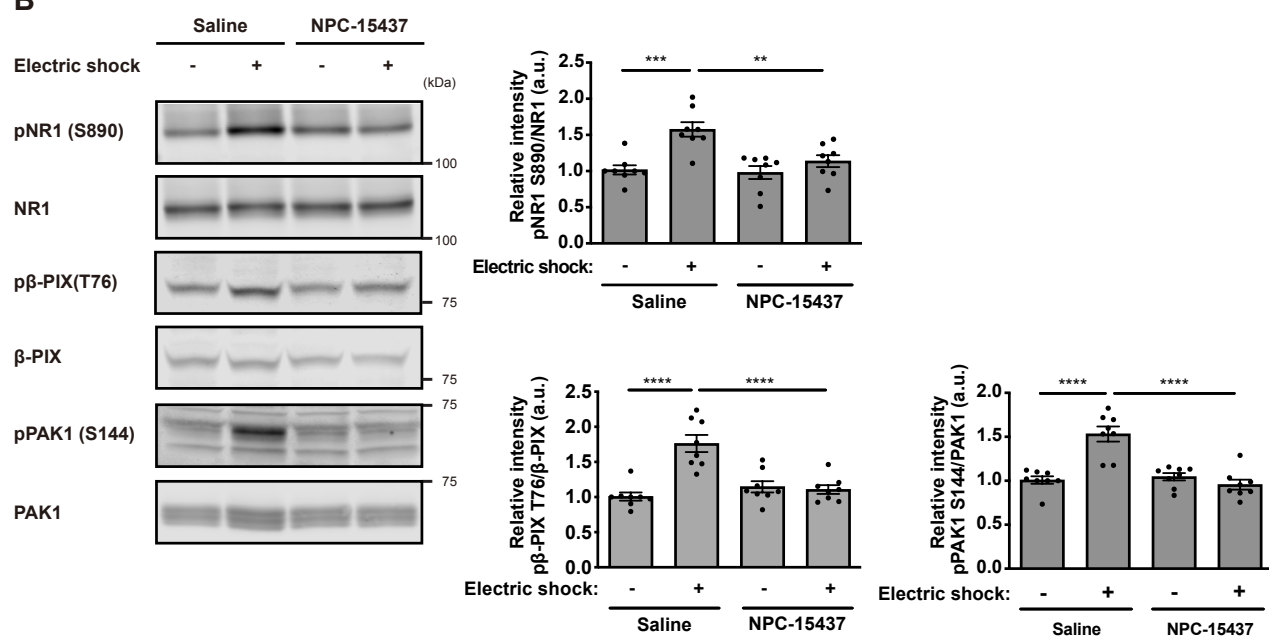

Supplement: Supplementary file 5 — Supplementary Figure 4 [file 41380_2022_1643_MOESM5_ESM.pdf]

Figure S5

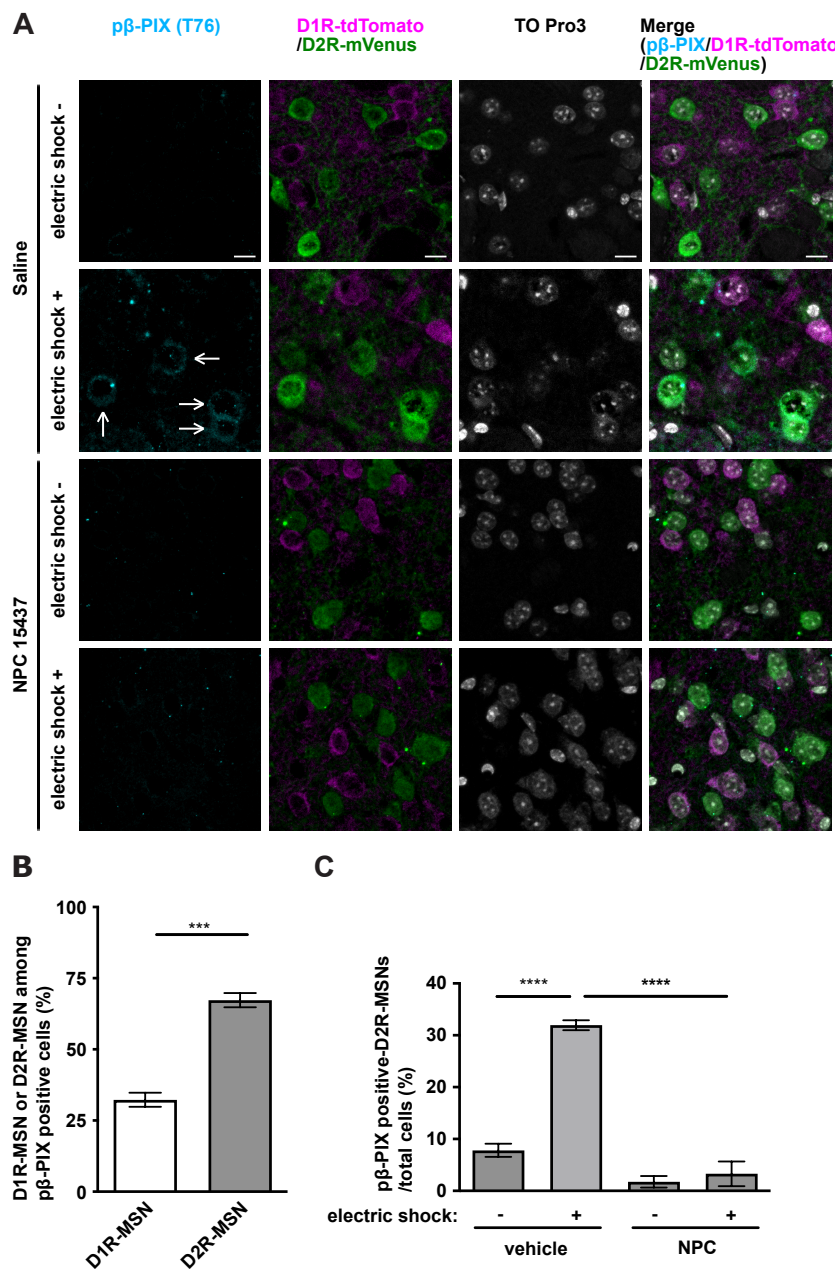

Supplement: Supplementary file 6 — Supplementary Figure 5 [file 41380_2022_1643_MOESM6_ESM.pdf]

Figure S6

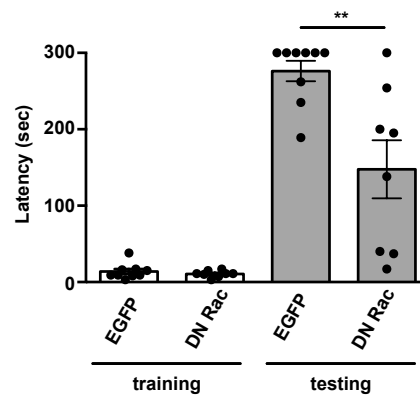

Supplement: Supplementary file 7 — Supplementary figure 6 [file 41380_2022_1643_MOESM7_ESM.pdf]

Figure S7

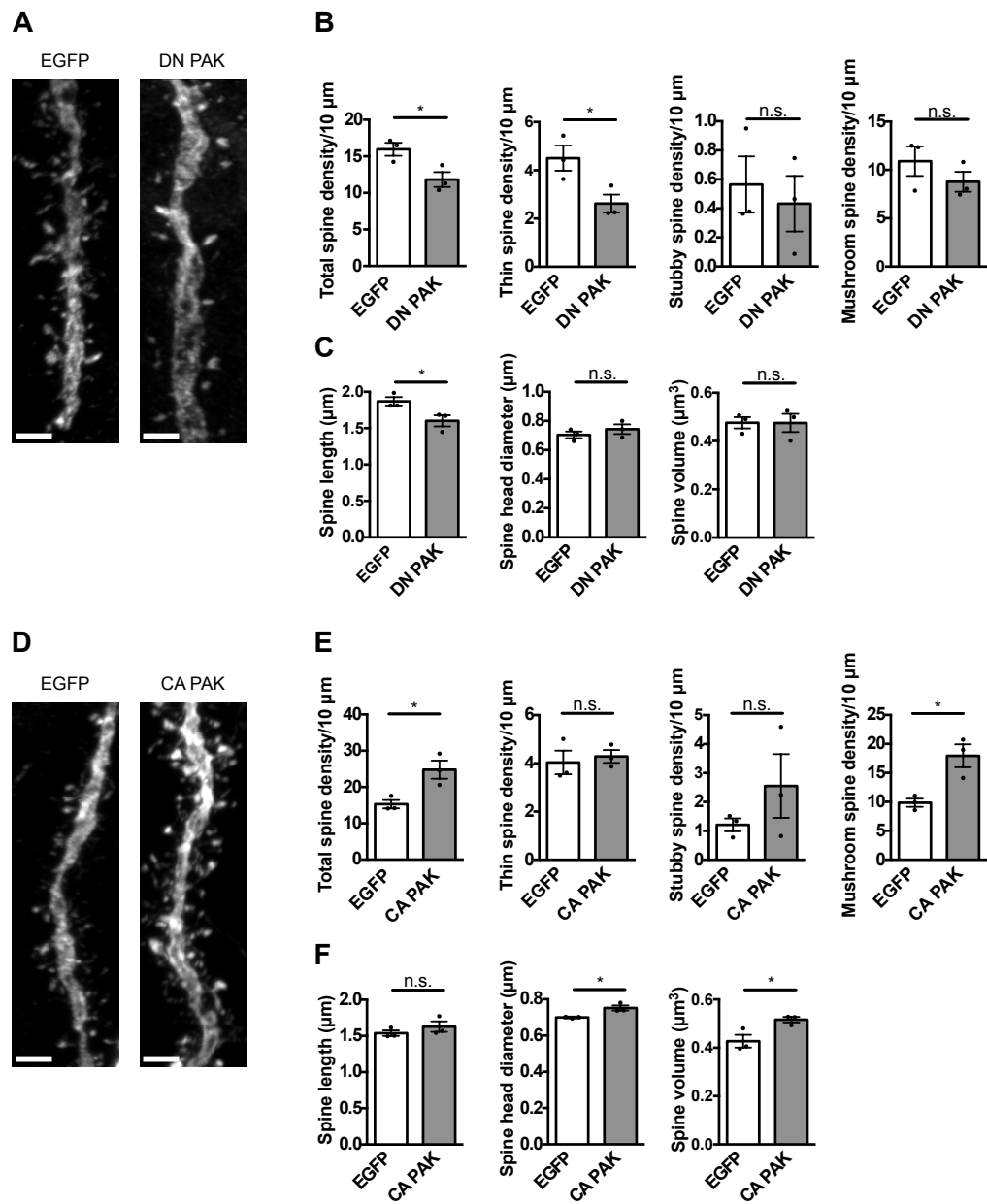

Supplement: Supplementary file 8 — Supplementary Figure 7 [file 41380_2022_1643_MOESM8_ESM.pdf]

Figure S8

**A**

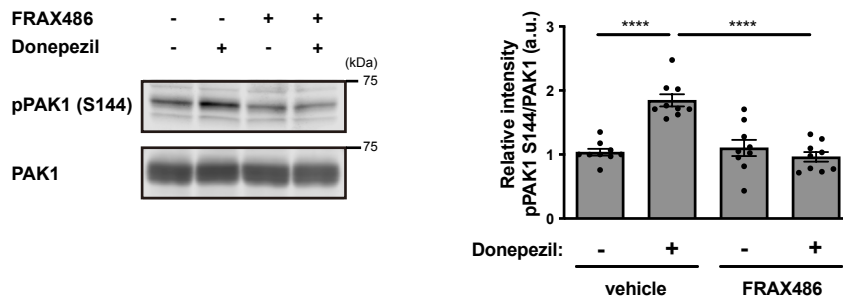

**B**

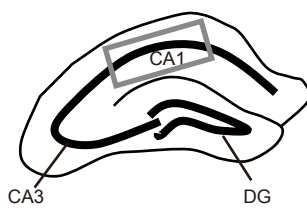

**C**

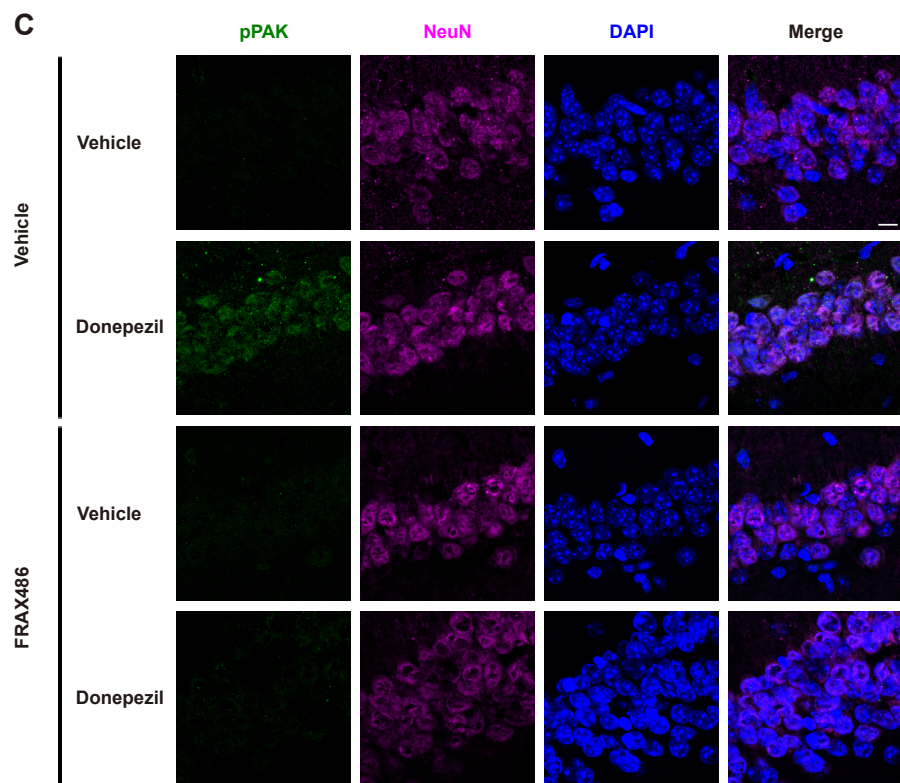

Supplement: Supplementary file 9 — Supplementary Figure 8 [file 41380_2022_1643_MOESM9_ESM.pdf]

Figure S9

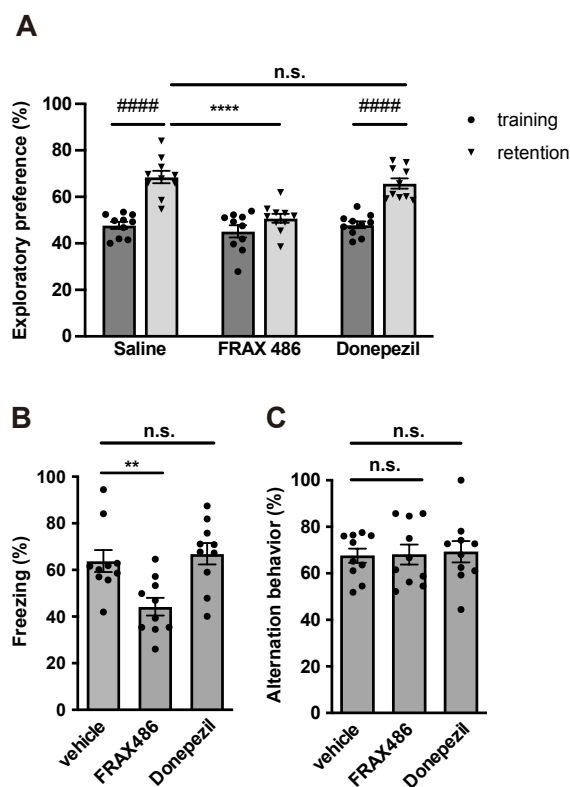

Supplement: Supplementary file 10 — Supplementary Figure 9 [file 41380_2022_1643_MOESM10_ESM.pdf]

Figure S10

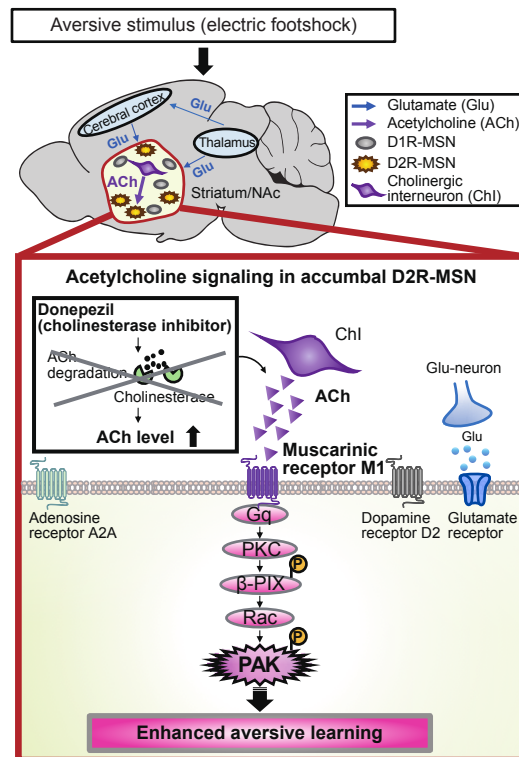

Supplement: Supplementary file 11 — Supplementary Figure 10 [file 41380_2022_1643_MOESM11_ESM.pdf]
